# Telomere attrition and dysfunction: a potential trigger of the progeroid phenotype in nijmegen breakage syndrome

**DOI:** 10.18632/aging.103453

**Published:** 2020-06-20

**Authors:** Raneem Habib, Ryong Kim, Heidemarie Neitzel, Ilja Demuth, Krystyna Chrzanowska, Eva Seemanova, Renaldo Faber, Martin Digweed, Reinhard Voss, Kathrin Jäger, Karl Sperling, Michael Walter

**Affiliations:** 1 Department of Human Genetics, Ruhr-University Bochum, Bochum, Germany; 2 Institute of Medical and Human Genetics, Charité - Universitätsmedizin Berlin, Berlin, Germany; 3 Institute of Clinical Chemistry, Red-Cross General Hospital, Pyongyang, Democratic People’s Republic of Korea; 4 Department of Endocrinology and Metabolism, Charité – Universitätsmedizin Berlin, Corporate Member of Freie Universität Berlin, Humboldt-Universität zu Berlin, Berlin Institute of Health, Germany; 5 Charité - Universitätsmedizin Berlin, BCRT - Berlin Institute of Health Center for Regenerative Therapies, Berlin, Germany; 6 Department of Medical Genetics, The Children’s Memorial Health Institute, Warsaw, Poland; 7 Department of Clinical Genetics, Institute of Biology and Medical Genetics, Second Medical School, Charles University, Prague, Czech Republic; 8 Center for Prenatal Medicine, Leipzig, Germany; 9 Integrated Functional Genomics, Interdisciplinary Center for Clinical Research, University of Münster, Münster, Germany; 10 Institute of Clinical Chemistry and Laboratory Medicine, University of Rostock, Rostock, Germany; 11 Institute of Laboratory Medicine, Clinical Chemistry and Pathobiochemistry, Charité – Universitätsmedizin Berlin, Corporate Member of Freie Universität Berlin, Humboldt-Universität zu Berlin, Berlin Institute of Health, Germany

**Keywords:** nijmegen breakage syndrome, telomere-position effect over long distances, alternative lengthening of telomeres, nibrin, DNA repair

## Abstract

Background: Nibrin, as part of the NBN/MRE11/RAD50 complex, is mutated in Nijmegen breakage syndrome (NBS), which leads to impaired DNA damage response and lymphoid malignancy.

Results: Telomere length (TL) was markedly reduced in homozygous patients (and comparably so in all chromosomes) by ~40% (qPCR) and was slightly reduced in NBS heterozygotes older than 30 years (~25% in qPCR), in accordance with the respective cancer rates. Humanized cancer-free NBS mice had normal TL. Telomere elongation was inducible by telomerase and/or alternative telomere lengthening but was associated with abnormal expression of telomeric genes involved in aging and/or cell growth. Lymphoblastoid cells from NBS patients with long survival times (>12 years) displayed the shortest telomeres and low caspase 7 activity.

Conclusions: NBS is a secondary telomeropathy. The two-edged sword of telomere attrition enhances the cancer-prone situation in NBS but can also lead to a relatively stable cellular phenotype in tumor survivors. Results suggest a modular model for progeroid syndromes with abnormal expression of telomeric genes as a molecular basis.

Methods: We studied TL and function in 38 homozygous individuals, 27 heterozygotes, one homozygous fetus, six NBS lymphoblastoid cell lines, and humanized NBS mice, all with the same founder *NBN* mutation: c.657_661del5.

## INTRODUCTION

Nijmegen breakage syndrome (NBS) was first described in 1981 in two patients in Nijmegen, in the east of the Netherlands [1]. It is characterized by chromosome instability associated with microcephaly, immunodeficiency, hypersensitivity to ionizing irradiation, and a high predisposition to cancer [2–4]. NBS also displays other symptoms of aging such as postnatal growth retardation, decline in mental function, gray hair, telangiectasias and café au lait spots. Nibrin, a DNA double-strand break (DSB) repair protein, is defective [5, 6], and more than 90% of NBS patients are homozygous for a founder mutation: c.657_661del5 [6–8]. Risk of death from lymphoma is elevated ~1000-fold; by the age of 20, more than 40% of patients have developed a malignant disease, predominantly of lymphoid origin. Even heterozygous carriers of the founder mutation have an increased risk of cancer [8]. In some areas of the world, the *NBN* gene became the most important cancer-predisposing gene [7].

Nibrin is part of the nibrin/Mre11/Rad50 (MRN) complex, which is involved in the repair of DNA double strand breaks (DSBs), the processing of DSBs in immune gene rearrangements, and meiotic recombination [9]. The important role of this complex in mediating the ATM-dependent repair of DSBs probably explains the predisposition to cancer and immunodeficiency in NBS. It is unclear, however, why the incidence of cancer is so much higher in NBS than in other genetic instability syndromes. Nibrin is multifunctional and may also play an important role in protecting the telomeres from inappropriate DNA repair. Telomeric DNA is an evolutionarily highly conserved repetitive sequence that plays a crucial role both in cellular senescence and in carcinogenesis. The exact role of the MRN complex and nibrin in particular in telomere homeostasis is not clear, even though there have been some groundbreaking experimental findings in recent years pointing to a key function in the response to dysfunctional telomeres [10, 11]. Unlike in *S. cerevisiae*, mammalian MRN is not required for association of telomerase to short telomeres [12]. However, experimental *in vitro* and animal data suggest that the MRN complex is required for activation of the ATM-dependent repair of dysfunctional telomeres, the resection of telomeric DNA to create the single-stranded 3’ overhang and for stabilization of telomeric T-loops, which is required for telomere replication and elongation [13, 14]. Telomeres recruit Mre11, phosphorylated nibrin, and ATM, which is important for protection and repair of telomeres [15, 16]. The MRN complex protects the leading-strand ends from non-homologous end joining (NHEJ) [17], whereby the telomeres seem to recruit Mre11, phosphorylated nibrin and ATM in every G2 phase of the cell cycle and thus promote the formation of a chromosome end protection complex and a localized DNA damage response [18]. It was proposed that nibrin is required for the proper assembly of the MRN complex, which includes ubiquitination of nibrin upon DSBs [19] and may indirectly influence ATM activation by Mre11 and Rad50 [20].

We therefore hypothesized that NBS is a telomeropathy [21, 22], and that telomere abnormalities may accelerate cancer manifestation. Shorter telomeres have been described in individual NBS cases, for both NBS lymphocytes [23, 24] and fibroblasts [25]. Nonetheless, a systematic investigation has not yet been carried out, and the importance of MRN in general and nibrin in particular for telomere length and function are unclear.

## RESULTS

### Telomere lengths in human NBS cells and in humanized NBS mice

**Figure 1:**
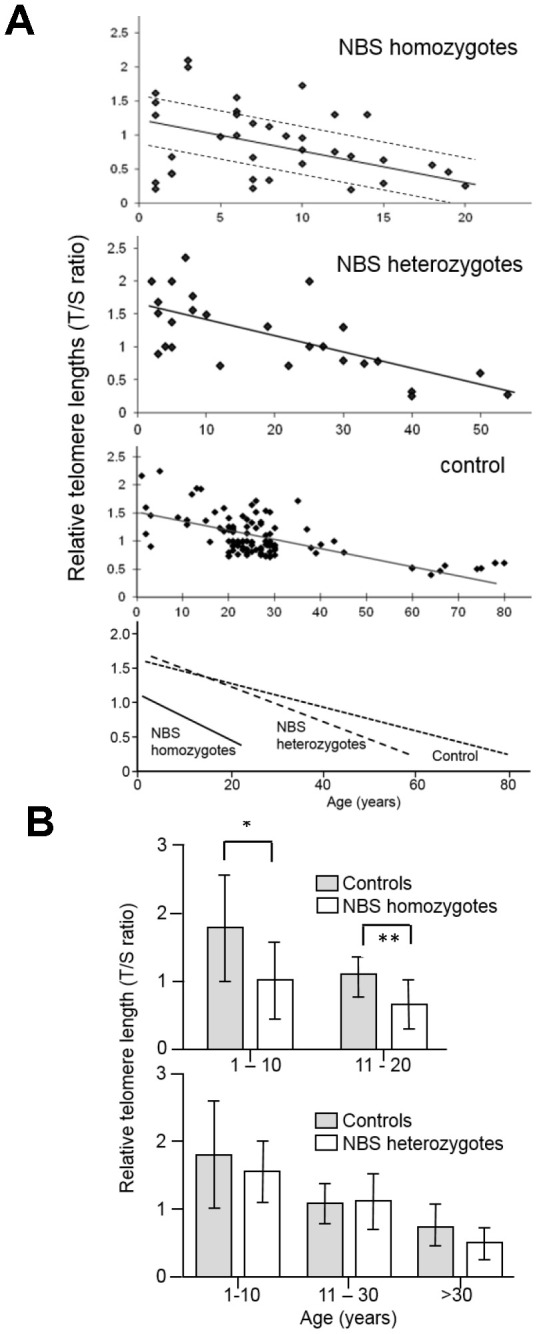
(**A**) Relative telomere length (TL) as a function of age in NBS homozygotes, heterozygotes, and control individuals. Relative TL (T/S ratio) was analyzed from blood samples of 38 NBS homozygotes, 27 NBS heterozygotes, and 108 control individuals by quantitative polymerase chain reaction (qPCR). The dashed lines separate the NBS homozygotes in those with long, medium, and short TL. Below: regression curves standardized for age. Original after thesis Raneem Habib [28]. (**B**) Comparison of TL, as analyzed by qPCR, of NBS homozygotes, heterozygotes, and controls. The comparison was made for age-matched groups (mean values and standard deviation). * indicates p<0.01; ** indicates p<0.001.

Relative leukocyte TLs of blood DNA from 38 NBS homozygotes, 27 heterozygotes, and 108 control individuals were measured by qPCR. The mean relative TL of NBS-homozygotes was ~40% shorter in two age-matched groups (1-10 and 11-20 years) than in the control group (p<0.05). We found mildly (~25%) reduced TLs in older NBS heterozygotes (>30 years old; p=0.1) but not in younger heterozygotes (Figure 1 and Supplementary Tables 1–3).

**Figure 2:**
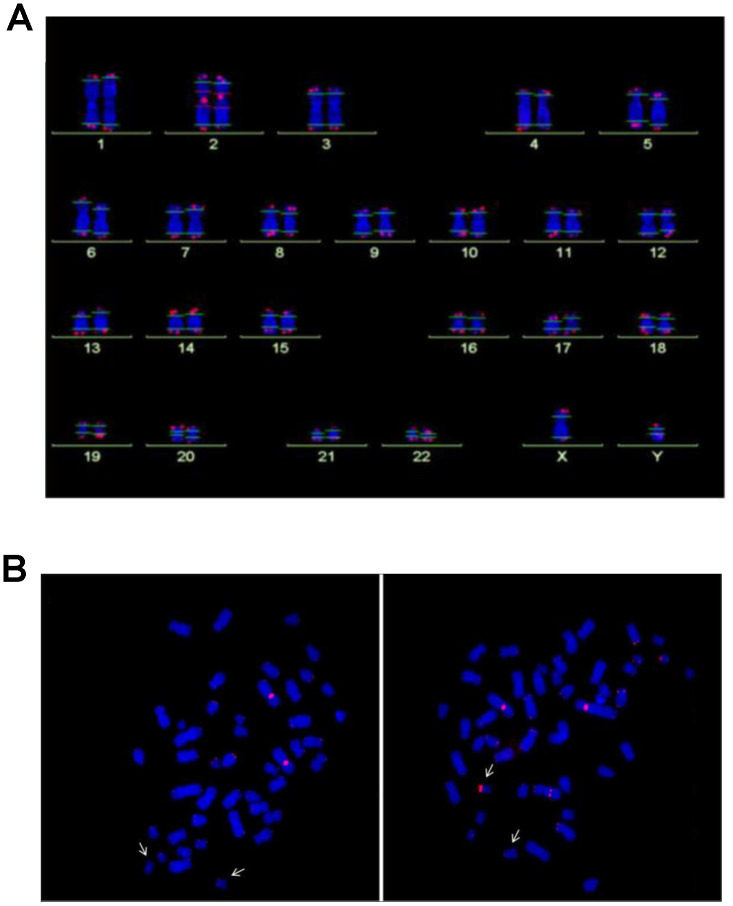
(**A**) Analysis of telomere length by Q-FISH. Normal karyogram with single telomeres stained for telomere repeats (Q-FISH) and a repetitive region in the centromeric region of chromosome 2. The horizontal lines overlaid on each chromosome define the measurement areas. (**B**) Metaphases of the NBS LCL 94P0307 after Q-FISH of the NBS cell line 94P0307 with very short telomeres and a telomere fusion. The arrows point to chromosomes 19. Left panel, Metaphase with weak telomerice fluorescence of both chromosomes 19; Right panel, Metaphase with one chromosome 19 with a brightly fluorescent telomere of the p-arm. The other bright signal is the reference region of chromosome 2. Original after thesis Raneem Habib [28].

**Figure 3:**
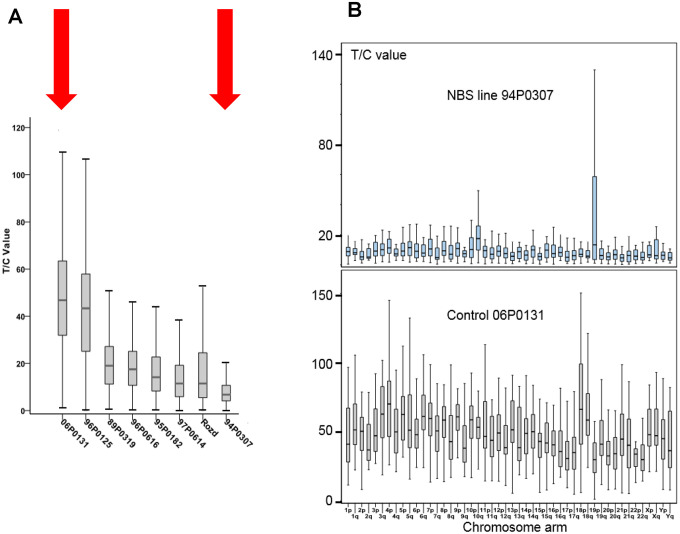
(**A**) Total telomere length (TL) analysed by Q-FISH. TL of six NBS lymphoblastoid cell lines and two control lines (06P0131 and 96P0125) was analyzed by Q-FISH. The boxplot presents the median, the minimum and the maximum T/C values. (**B**) Individual TLs of the NBS line 94P0307 and the control line 06P0131 (red arrows in Figure 3A) analyzed by Q-FISH of 15 metaphases. The boxplot presents the median, the minimum and maximum T/C values. Note the huge variability in TL of the short arm of one chromosome 19 (19p). Original after thesis Raneem Habib [28].

For Q-FISH analysis, six NBS lymphoblastoid cell lines derived from three individuals with extremely short survival after cancer manifestation (<3 years), and from three individuals with remarkably long survival (>12 years) were analyzed. All six lymphoblastoid cell lines had TLs that were markedly reduced (by ~60-75%) relative to healthy controls (p<0.05). Rather short telomeres were found on chromosomes 17, 19, and 20, but this pattern is also a characteristic of normal diploid cells [26], and no preferred shortening of a particular chromosome was observed (Figures 2 and 3).

A positive correlation was found between the TL measured by Q-FISH and the TL measured by qPCR (r=0.96); (Supplementary Figure 1). Regression analysis was applied to transform the qPCR data into absolute TLs. In the formula y=3.4(X)+10.11, y represents the predicted TRF (Terminal Restriction Fragment) value in kb, X represents the qPCR (T/S) value. The qPCR data and the TRF values showed a moderate correlation (r=0.64; Supplementary Figure 2).

**Figure 4:**
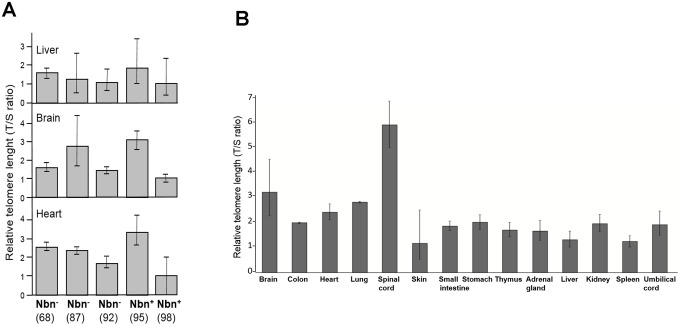
(**A**) Relative telomere length (TL) of three tissues of humanized Nbs mice as estimated by qPCR. Relative TL (T/S ratio) in brain, heart, and liver tissues of humanized Nbs mice (*Nbn^-/-^NBN^del^*, in the figure labelled Nbn^-^) 87, 68 and 92 (age: 32, 41, and 39 days) and humanized wild-type mice (*Nbn^-/-^NBN^+^*, in the figure labelled Nbn^+^): 95 and 98 (age: 32 days) estimated by qPCR. (**B**) Relative TL of an NBS fetus. Relative TL ±SD (T/S ratio) in 14 different tissues of a 32 week old NBS-fetus estimated by qPCR. Original from [28].

A rather uniform TL distribution has been described for most fetal tissues of healthy individuals [27]. In contrast, we found considerable differences in TLs between different fetal tissues: spinal cord and brain tissues had the longest telomeres, while fibroblasts and skin had the shortest, suggesting substantial telomere attrition in these tissues before birth (Figure 4B).

The common human NBN mutation c.657_661del5 is hypomorphic, allowing low-level, functionally relevant, truncated nibrin protein to be formed through an alternative initiation of translation. This is relevant because a loss of function leads to early embryonic lethality in mice [29]. Nbn-/- mice with the human NBS allele display most of the human NBS characteristics with one exception: humanized NBS mice are not prone to early tumorigenesis [29]. The humanized mice analyzed in this investigation expressed the human *NBN* gene, generated by the introduction of the human allele including the 5 bp deletion into *Nbn*-deficient mice (*Nbn^-/-^NBN*^del5^). The T/S ratio of control and *Nbn*-deficient mice showed some variability but we did not find significant differences in the TLs between the mice with the NBN founder mutation and the mice with the wild type allele (Figure 4A).

### Genetic instability in human NBS cells

The NBS cell lines exhibited spontaneous aberrations, such as chromatid breaks and translocations, as previously described [1–6]. The NBS cell lines in this study displayed a markedly increased rate of chromatid breaks after irradiation (Supplementary Table 4). In one cell line (94P0307) we did find telomere fusions that had not been described in previous NBS studies. All individual telomeres of this line were shorter than those of the control line 06P0131 with one exception: the telomere of the p arm of one chromosome 19, which showed an enormous variability in length (Figure 2B; Figure 3 and Supplementary Table 5), due to cellular mosaicism. This telomere was brightly fluorescent in approximately 70% of the metaphases with decreasing tendency during cultivation. The difference in relative T/C values was approximately 11:1 for the two chromosomes 19 (Supplementary Table 6). The expression of *hTERT* was weak in this and all other lymphoblastoid lines analyzed, close to the lower detection limit (Supplementary Figure 3), pointing to alternative lengthening of telomeres (ALT) as the most likely mechanism of telomere elongation in this cell line. Telomere dysfunction–driven genome damage associated with chromosomal break-fusion-bridge cycles and double-strand breaks are frequent in ALT cell lines [30]. In this context, we were interested that the cell line 94P0307 had twice as many aberrations (chromatid breaks and translocations) compared to the other two cell lines with long survival (Supplementary Table 4 and Supplementary Figure 4).

### Abnormal regulation of telomeric genes in NBS cells

We investigated possible functional consequences of telomere elongation on 19p and of the expression of telomeric genes *per se*. Telomeres may loop to specific loci to regulate gene expression; this process is called TPE-OLD (telomere position effect over long distances) [31]. Individual genes or gene regulators close to the telomeres are silenced in young cells (with long telomeres) and become expressed when telomeres are short. Re-elongation of short telomeres in cells by exogenous expression of the *hTERT* gene (active telomerase) normally results in reorganization of functional telomeres and expression patterns similar to those in young cells with long telomeres, up to 10-15 Mbp away from the telomere [31]. We were interested to know if long 19p telomeres might influence the mRNA expression of telomeric genes. Using Affymetrix cDNA microarray analyses and fibroblasts from healthy volunteers and Hutchinson Gilford progeria (HGP) patients we identified ten genes on 19p that were differentially regulated at the level of mRNA in pre-senescent cells (with short telomeres) and in *hTERT* immortalized cells (with long telomeres) and that were not differentially regulated by UV-B irradiation as a model for stress-induced senescence (Supplementary Table 7 and Supplementary Figures 5–8).

**Figure 5:**
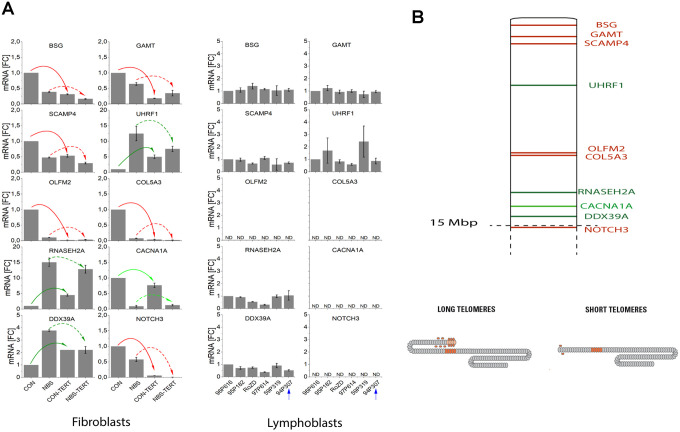
(**A**) mRNA expression of TPE-OLD candidate genes in cells with normal/short telomeres and artificially elongated telomeres in the presence of hTERT. qPCR analysis was performed in a healthy human fibroblast cell line, a NBS fibroblast cell line, and in all 6 available lymphoblastoid cell lines (LCLs). Total mRNA was extracted from proliferating fibroblasts and from the same cell lines proliferating with experimentally elongated telomeres, after immortalization with *hTERT*. In LCLs, mRNA from the 6 different donors was compared. One of these cell lines (94P307) showed extremely elongated telomeres on p19. The genes were identified as TPE-OLD candidates by use of Affymetrix gene chip experiments in independent cell lines from healthy controls and HGP patients (as described in Supplementary Tables 7–8 and Supplementary Figures 5–8). All values were normalized to the level (=1-fold) of mRNA in unmodified and pre-senescent control fibroblasts. Each assay was performed in triplicate. Red arrows mark the genes with attenuated regulation in NBS (short telomeres in pre-senescent vs. long telomeres in *hTERT* infected cells). Green arrows mark the genes with reversed regulation in NBS (dark green: downregulated in pre-senescence in controls; light green upregulated). The blue arrow marks the LCL with extremely long telomeres on 19p. *BSG*: basigin; *GAMT*: guanidinoacetate methyl-transferase; *SCAMP4*: secretory carrier membrane protein 4; *OLFM1*: olfactomedin 1; *UHRF1*: ubiquitin like with PHD and ring finger domains 1; *COL5A3*: collagen type V alpha 3 chain; *RNASEH2A*: ribonuclease H2 subunit A; *CACNA1A*: calcium voltage-gated channel subunit alpha1 A; *DDX39A*: DExD-box helicase 39A; *NOTCH3*: Notch receptor 3. ND, non detectable (mRNA quantifications with Ct values above 35). Borderline Ct values were detected for NOTCH3 for the LCL 95P182 (mean Ct = 34.9) and 59P319 (mean Ct = 34.6), not shown in the figure. (**B**) TPE-OLD concept. Telomeres loop to specific loci to regulate gene expression, a process termed telomere position effect over long distance [31]. The effect may likely extend to a distance of at least 10-15 Mbp from the telomere. The marked TPE-OLD gene candidates on 19p were investigated in experiments shown in Figure 5A: *BSG*, *GAMT*, *SCAMP4*, *OLFM2*, *COL5A3*, *CACNA1A*, *NOTCH3* (upregulated in pre-senescent cells), and *UHRF1*, *DDX39A* and *RNASEH2A* (downregulated in senescent cells). TPE-OLD genes may form clusters with reverse regulation in NBS (green) further away from the telomere.

As shown in Figure 5 and Supplementary Table 8, differential regulation in array experiments was confirmed by qPCR for all ten genes in the healthy control fibroblasts. The extremely elongated telomere of 19p in the lymphoblastoid cell line did not show any suppressive or enhancing effect on mRNA level (Figure 5). By contrast, the expression of all TPE-OLD candidate genes was markedly altered in pre-senescent NBS fibroblasts. We observed lower mRNA levels for *BSG* (-61%), *GAMT* (-35%), *SCAMP4* (-52%), *OLFM2* (-90%). *COL5A3* (-92%), *CACNA1A* (-91%) and *NOTCH 3* (-42%), and we observed increases for *UHRF1* (12.5-fold), *RNASEH2A* (15.1-fold), and *DDX39A (3.8*-fold) in pre-senescent NBS compared to pre-senescent control fibroblasts. The telomere-dependent regulation was even reversed for *UHRF1*, *RNASEH2A*, *CACNA1A* and *DDX39A*. All TPE-OLD candidates encode proteins involved in senescence or cell growth. *BSG* encodes the metalloproteinase EMMPRIN that may trigger matrix metalloprotease and cytokine production and plays an important role in heart remodeling in aging mice [32]. The *GAMT* gene product guanidinoacetate methyltransferase catalyzes creatine synthesis, which may help to replenish cellular ATP [33], possibly for senescence-associated secretory phenotype (SASP) [34]. *SCAMP4* gene product secretory carrier membrane protein is a direct player in SASP [35]. The *OLFM2* gene product olfactomedin 2 is an age-dependent regulator of cell differentiation and regulates axonal growth [36]. *COL5A3* induces collagen synthesis and is involved in age-dependent tissue remodeling [37]. *CACNA1A* regulates calcium entry age-dependently and plays a role in neurodegeneration [38]. *NOTCH3* functions as a tumor suppressor by controlling p21-mediated cellular senescence [39]. By contrast, *UHRF1* is a negative regulator of senescence. Cellular senescence can be induced by phosphorylation and inactivation of UHRF1 [40]. *RNASEH2A* enhances migration and invasion and may play a role in cancerogenesis [41]. *DDX39A* is a RNA helicase and Telomeric Repeat Factor 2 (TRF2)-interacting protein with suspected roles in both cancer and longevity [42]. Altogether, these data suggest abnormal regulation of telomeric aging genes in NBS cells.

### Relationship between telomeres length, disease progression, and cell pathology

There was no significant correlation between the TL and either the age at cancer manifestation or age at death (Table 1, Supplementary Table 9). This result was surprising insofar as we expected a worse phenotype in patients with short telomeres. Thus we explored the possibility that short telomeres may have stabilized the phenotype in a senescence-like state in all or in individual patients. To test this possibility, we measured caspase 7 activity as a surrogate marker for apoptosis. Senescent fibroblasts resist apoptosis by downregulating caspases. As shown in Table 2, caspase activity was significantly higher in the three cell lines derived from patients with shorter survival and longer telomeres compared to those with longer survival and shorter telomeres (2.8-fold; P<0.05, 48h after 10 mg/ml bleomycin). The cell line 94P0307 with the shortest telomeres displayed an extremely low (almost undetectable) caspase activity. Moreover, the cell lines derived from patients with shorter survival displayed on average twice as many chromatid breaks as the cells from patients with long survival (Supplementary Table 4). Altogether these data point to a more stable phenotype with a higher “degree” of senescence in cells from patients with long survival.

**Table 1:** Relationship of telomere length of NBS homozygotes with age at cancer manifestation and age at death.

**Telomere length**	**Origin**	**ID/DNA**	**Cancer**	**Ig Status**	**Age at cancer manifest.**	**Mean ± SD**	**Age at death**	**Mean ± SD**
Long*	Po	94P629	B-NHL	IgA+IgG↓	9	9.2 ± 3.49	9	15.80 ± 9.04
	Po	97P614	B-NHL	normal	7		19	
	Po	94P307	DLBCL-1	normal	11		29	
		DLCBL-5		27			
	Po	8294	T-NHL	unknown	14		16	
	Po	98P222	Pre-B-ALL	unknown	5		6	
Inter-mediate*	Ge	94P126	NHL	unknown	13?	19.0 ± 10.04	19?	19.71 ± 9.78
	Po	97P081	T-NHL	IgA+IgG↓	24		24	
	Po	94P251	HL	IgA+IgG↓	12		14	
	Po	94P548		IgA+IgG↓			8	
	Po	5567	T-NHL	unknown	34		34	
	Po	96P551		normal				
	Cz	5431	NHL	unknown	24		29	
	Cz	7822	NHL	unknown	7		10	
Short*	Po	97P610	NHL-Burkitt-like/TLBL	IgA ↓	6	14.4 ± 12.90	19	15.83 ± 12.16
					17			
	Po	96P473		IgA+IgG↓				
	Po	97P229	B-NHL	IgA+IgG↓	4		6	
	Po	97P751	Cause of death unclear	unknown			3	
	Po	95P185		IgA+IgG↓				
	Po	95P182	B-NHL	IgA+IgG↓	8		10	
	Po	94P195	T-NHL Precursor TLBL	normal	19		21	
	Po	94P192	TLBL/ALL?	IgG↓	35		36	

*Age-adjusted Po, Poland; Ge, Germany; Cz, Czech Republic.

**Table 2:** Analysis of caspase-7 and ATM phosphorylation after bleomycin treatment of six NBS lymphoblastoid cell lines with the absolute longest (above) and absolute shortest (below) telomere lengths.

	**Survival (y)**	**Caspase-7 activity after bleomycin treatment (10 μg/ml)**				**pATM/ATM level after bleomycin (1h)**		
		0h	12h	24h	48h	0 μg/ml	10 μg/ml	30 μg/ml
89P0319 ♀	0	0.65±0.26	0.77±0.22	0.78±0.17	0.90±0.13	0,17±0,05	0,56±0,01	0,74±0,03
96P0616 ♀	0,2	0.17±0.01	0.24±0.01	0.41±0.05	0.90±0.08	0,07±0,05	0,24±0,06	0,47±0,06
95P0182 ♀	2,8	0.17±0.14	0.25±0.15	0.46±0.11	0.65±0.10	0,04±0,03	0,28±0,07	0,53±0,06
		**0.33±0.28**	**0.42±0.30**	**0.55±0.20**	**0.82±0.14**	**0,09±0,07**	**0,36±0,17**	**0,58±0,14**
97P0614 ♂	>12	0.13±0.03	0.16±0.05	0.36±0.09	0.49±0.05	0,04±0,02	0,16±0,07	0,22±0,08
RoZd ♀	>12	0.13±0.08	0.15±0.01	0.27±0.01	0.36±0.05	0,06±0,05	0,30±0,09	0,39±0,04
94P0307 #x2642;	>12	0.01±0.01	0.02±0.01	0.02±0.01	0.03±0.02	0,11±0,08	0,29±0,06	0,45±0,02
		**0.09±0.07**	**0.11±0.08**	**0.22±0.18**	**0.29±0.24**	**0,07±0,04**	**0,25±0,08**	**0,35±0,12**
		P=0.22	P=0.16	P=0.09	**P=0.03**	P=0.62	P=0.38	P=0.10

Each experiment was repeated three times; Mean ± SD.

There is a close functional link between ATM and nibrin [43]. As shown in Table 2, the radiomimetic bleomycin induction of ATM-Ser 1981 phosphorylation was not significantly different among cell lines with short or long survival rates. Also, the cell line with low caspase 7 activity had normal ATM function.

## DISCUSSION

### Accelerated telomere attrition and telomere dysfunction in NBS cells

The main finding of this work is the stronger than expected telomere shortening and functional restriction in NBS patients. NBS homozygotes showed significantly shorter TL in all age groups. TL was reduced in homozygous patients by ~40% (qPCR). TL was ~25% shorter in NBS heterozygotes older than 30 years. The latter result was borderline significant (p=0.1) but remarkable in view of the lower age in NBS heterozygotes (42 y vs. 57 y in controls). Even stronger differences in TL were observed with qFISH (60-70%) in a subset of six homozygous NBS patients.

The variability of TL depending on the method suggests that telomeric and subtelomeric sequences are affected. We found only a moderate correlation between qPCR and TRF values, which is plausible insofar as the localization of the subtelomeric region included in the measurement is variable in TRF analysis based on the restriction enzymes used. Similar observations (with comparable correlation coefficients) have been made in other studies [44]. The advantage of the TRF method is the measurement of TL in absolute values (bases); on the other hand there is the disadvantage of being dependent on restriction enzymes with interindividual and intersexual biases [45]. Using qFISH rather short telomeres were found on chromosomes 17, 19, and 20. Yet this pattern was very similar to that of normal diploid cells [26].

In one NBS lymphoblastoid cell line (94P0307), we found striking telomere fusions with extreme telomere elongation in cells with very low *hTERT* expression. There is evidence that in humans the *hTERT* gene is close to the telomere and influenced by TPE-OLD [46]. However, silencing of *hTERT* in this cell line is unlikely because the *hTERT* gene is located on chromosome 5p and the extremely elongated telomere is located on chromosome 19p. We did not search for c-circles and ALT-associated promyelocytic leukemia protein (PML) bodies in this investigation. However, we had some indirect evidence for possible ALT involvement. Telomere dysfunction–driven genome damage associated with chromosomal break-fusion-bridge cycles and double-strand breaks are frequent in ALT cell lines [30], and were also observed in this cell line. This is remarkable insofar as ALT normally requires the activity of the Mre11/Rad50/nibrin recombination complex [14, 47, 48]. However, there are several ALT mechanisms [14], and ALT may have occurred by homologous recombination and telomere-sister chromatid exchange [14]. Altogether, these *in vitro* experiments are consistent with both the proposed function of nibrin in telomere protection [18–20] and with the occurrence of cancer forms with high ALT prevalence in NBS [49].

Telomere extension was possible with telomerase in cultivated control and NBS fibroblasts. We were able to find expected changes on mRNA levels in control fibroblasts for all TPE-OLD candidate genes investigated on 19p. But we did not find expression changes of telomeric genes for the lymphoblastoid cells with abnormal 19p elongation; the TL-dependent regulation of the genes *BSG, GAMT, SCAMP4,* *OLFM2, COL5A3,* and *NOTCH 3* was attenuated in NBS fibroblasts, and it was reversed for the genes *UHRF1, RNASEH2A, CACNA1A* and *DDX39A*. These data show that NBS cells are still capable of elongating telomeres. Yet functionally abnormal telomeres may arise.

### Possible causes for accelerated telomere attrition in NBS cells

The NBN mutation c.657_661del5 may affect a variety of functions leading to disturbed telomere repair and accelerated telomere attrition. The MRN complex is required for activation of the ATM-dependent repair of dysfunctional telomeres, the resection of telomeric DNA to create the single-stranded 3’ overhang and for stabilization of telomeric T-loops [13, 14]. The Mre11-nibrin interaction required for ATM activation [20] may be affected as well as nibrin ubiquitination [19] and/or phosphorylation [18], which is important for cell cycle dependent telomere conservation and repair [18]. In accordance with current concepts (with nibrin as a co-factor for MRN complex assembly and a more indirect activation of ATM [18–20]) ATM phosphorylation was not impaired in our experiments, which does not point to a direct functional interference with ATM activation.

Increased oxidative stress has previously been described for NBS cells [50, 51]. The observation that G:C>A:T is the most frequent spontaneous base mutation in NBS [52] is of interest insofar as the telomeric TTAGGG sequence is sensitive to oxidative modifications and single-strand breaks [53]. Thus a high vulnerability of telomeres to oxidative damage may contribute to accelerated telomere attrition. The occurrence of ALT cells, and persistent DNA replication stress may additionally lead to spontaneous mitotic telomere synthesis, a potential driver of genomic duplications in cancer [14]. Our findings in fetal tissue suggest that substantial TL attrition may occur before birth in NBS homozygotes, which cannot be explained by the “end replication problem” alone. There were considerable differences in TLs between different fetal tissues. This is in sharp contrast to observations from healthy fetuses showing a very uniform TL in all tissues at all gestational ages [27].

### Possible implications of telomere attrition for cancerogenesis

Genetic instability and impaired telomeric repair may synergize with telomere attrition and dysfunction to explain the extremely high cancer incidence in NBS. The “dose dependency” of telomere attrition on the phenotype also account for this possibility. Homozygote NBS patients with marked telomere attrition develop cancer at young age. Heterozygote patients have an increased cancer risk at higher age, and humanized NBS mice with normal TL did not display early tumorigenesis, but showed all other signs of NBS [29]. The higher TL reserve in mice [53, 54] may explain why we observed no significant reduction in TL in the NBS mice. Overall, these data are consistent with epidemiological studies that have demonstrated a strong inverse relationship between TL and cancer incidence in the general population [55–57].

A possible explanation for the high lymphoma incidence in NBS could be the high rate of somatic recombination with associated hypermutability. This hypermutability is required for the extreme degree of somatic recombination of the immunoglobulin and T-cell receptor genes necessary for the vast repertoire of antibodies and T cell receptors [56]. On the other hand, the almost inevitable development of T or B cell lymphoma during childhood and the extreme preference over other types of cancer is striking. Two key steps are important for tumorigenesis: increased mutation rate and clonal selection with a growth advantage. The unique combination of hypermutability and extreme telomere attrition may have contributed to the high incidence of lymphomas at young age. In this context, it is noteworthy that all suggested TPE-OLD genes on p19 are functionally involved in cell senescence or cell growth and that all these TPE-OLD candidates were dysregulated in the NBS fibroblast cell line. Genes with suggested functions in senescence were suppressed, whereas growth promoting genes were rather increased on mRNA level (Figure 5 and Supplementary Table 8). Even if we had not the opportunity to show this dysregulation in cultivated (Epstein-Barr virus transformed) lymphoblastoid cell lines it is possible that TPE-OLD dysregulation favors a growth advantage *in vivo*, which synergizes with the extreme hypermutability and may explain the dramatically increased risk for developing lymphoma to over 1000-fold in NBS, which was not found in any other disease, including other DNA repair syndromes [56].

### Possible implications of telomere attrition for tumor suppression

We did not find an inverse correlation between TL and age of cancer onset. A combination of the TL, the degree of telomere damage, the number of critically short telomeres per cell, and other individual factors may also influence cancer onset. Alternatively, the deleterious effect of TL attrition is to some extent superimposed by some other more beneficial effect. In this context, we found it interesting that individual patients survived their disease for more than a decade. Cultivated lymphoblastoid cells derived from NBS patients with long survival times (>12 years) displayed shorter telomeres and lower caspase 7 activities, compared to cells derived from patients with short survival times (<3 years), suggesting low apoptosis rates and/or increased senescence rates with (at least in these individual patients) effective tumor suppression. The limit on cellular proliferation for cells with short telomeres is considered an initial block to oncogenesis [58]. Data may point to a direct protective effect of short telomeres in these patients.

**Figure 6:**
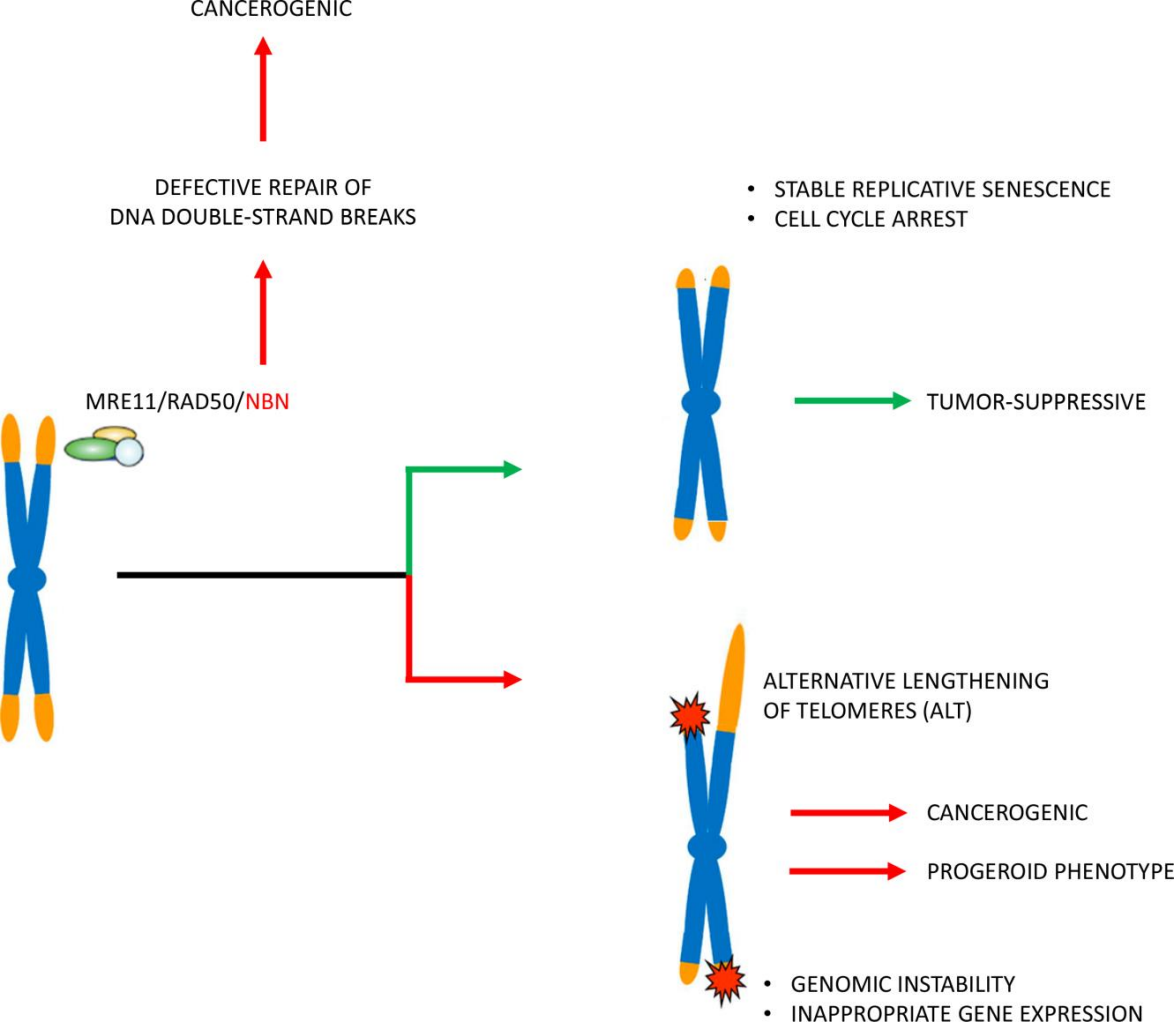
**Pathophysiological concept.** The patients’ cells in Nijmegen Breakage Syndrome have markedly reduced telomere lengths. Telomere attrition may induce genetic instability and alternative telomere elongation and thus enhance the cancerogenic effect (that is induced by defective repair of DNA double-strand breaks by non-homologous end joining) and also contribute to a progeroid phenotype. Telomere attrition may, however, also mitigate the clinical phenotype by inducing stable replicative senescence and cell cycle arrest. The latter is indicated by cells from patients with long tumor survival times with very short telomeres and little apoptosis.

These results are important to a better understanding of the role of telomere attrition in carcinogenesis *per se*. It is generally accepted that telomere attrition drives genomic instability and that the subsequent acquisition of *hTERT* expression and telomerase activity is involved in cancer immortality [59]. This model is supported by clinical findings showing that short telomeres are an important risk factor for malignant transformation [57]. On the other hand, longer telomeres can be a predictor for poor outcome after a tumor has developed [60]. This, at first glance, contradictory observation is usually attributed to higher telomerase activities (and thus a higher “degree” of immortality) in tumor patients with poor outcome. However, other findings indicate that the activation of telomerase may occur early in tumor development [59, 61]. The exact temporal sequence of telomerase activation, telomere shortening and malignant transformation is not clear. The data presented here illustrate the double-edged sword of telomere shortening in NBN founder mutation carriers with lymphoid malignancies (and thus confirm the current concept) but also show an alternative explanation for the correlation between TL and tumor progression: lower apoptosis and/or increased replicative senescence rates due to short telomeres and thus relative protection against new tumors (summarized in Figure 6). This may have important therapeutic implications. For example, hematopoietic stem cell transplantation (HSCT) appears to be a promising therapeutic strategy for some but not all NBS patients [62]. From a therapeutic point of view, our data suggest that telomere stabilization should indeed be the goal for NBS (and possibly other tumor) patients *before* cancer onset, but after cancer has already developed, “keeping telomeres short” would probably be more useful. Considering new therapeutic options for influencing telomeres [58] these findings could become clinically relevant for this and other cancer-predisposing mutations with involvement of telomere attrition and/or dysfunction.

Estimating the relative contribution of telomere shortening to cancerogenesis is difficult, especially in view of the bifunctional role of telomere shortening. The DNA repair defect has likely a major contribution to cancerogenesis. According to recent recommendations for a novel nomenclature [21, 22], NBS is clearly a secondary telomeropathy. Our data do not currently warrant the classification of the disease as a classical “impaired telomere maintenance spectrum disorder” (ITM). Secondary telomeropathies include diseases in which it is not clear to what extent the telomere dysfunction is the cause of the pathology.

### Possible implications of telomere attrition for the progeroid phenotype

NBS belongs to the progeroid syndromes and patients have symptoms of aging such as decline in mental function, gray hair, teleangiectasia and café au lait spots. It has been shown that transient introduction of telomerase mRNA into progeria cells improves many of the hallmarks of this disorder [63]. It is assumed that telomere dysfunction in Hutchinson Gilford progeria and likely other telomere syndromes is a causative molecular mechanism of pathogenesis [64]. The data presented here expand this concept and show that abnormal TPE-OLD may contribute to the progeroid phenotype in NBS. Using independent methods comparing replicative, stress-inducible and *hTERT* immortalized cells, we identified ten TPE-OLD candidate genes with significant TL-dependent expression changes. Seven of the TPE-OLD candidate genes were upregulated in aged healthy fibroblasts and are involved in senescence pathways or have some functional relation to aging. Three of the genes had rather growth promoting function and were suppressed in normal fibroblasts on mRNA level (Figure 5 and Supplementary Table 8). The TL-dependent response was attenuated for all seven upregulated genes in NBS fibroblasts and was reversed in all three growth promoting genes, suggesting that the normal senescence program is disturbed in these cells and shifted to a rather growth promoting expression pattern.

Thus coordinated regulation of telomeric genes could be important for (patho)physiology. We postulate that the progeroid phenotype in NBS is intimately influenced by TPE-OLD. In the age-associated genetic disease facioscapulohumeral muscular dystrophy *SORBS2* transcription is altered by a telomeric 4.8-Mb loop in patients´ myoblasts. *SORBS2* is normally up-regulated by maturation/differentiation of skeletal muscle and is misregulated by TPE-OLD-dependent variegation in myoblasts [65]. NBS is a secondary telomeropathy in which telomere dysregulation occurs at multiple sites. We cannot entirely exclude that some changes on mRNA level occurred secondarily as part of an adaptation mechanism. However, the extremely accelerated telomere attrition is a plausible explanation for abnormal TL-dependent regulation in NBS cells and modulation of the clinical phenotype. A role in pathogenesis is also supported by the significantly milder clinical phenotype in NBS mice with significantly longer telomeres. Humanized NBS mice have less severe immune system defects and are not markedly prone to malignancy.

The pathophysiological role of short telomeres in diseases with short telomeres is mostly attributed to replicative exhaustion, increased DNA damage response, genetic instability and missing scavenger for oxygen radicals. The studies presented here show another mechanism that could be effective independent from DNA damage response and long before the onset of senescence: an imbalance or dysfunction in telomere position effect.

All progeroid syndromes can be classified into two categories: those caused by alterations in components of the nuclear envelope; and those caused by mutations in components of the telomerase complex. Short telomeres have now been described for almost all progeroid syndromes with mutations in genes involved in DNA-repair pathways, including Rothmund-Thomson syndrome, Werner Syndrome, Dyskeratosis congenita, Ataxia teleangiectasia, Ataxia teleangiectasia like syndrome, Bloom syndrome, and NBS [21, 22]. The progeroid syndromes with short telomeres resemble each other in many aspects and short telomeres are a potential cause or co-factor for symptoms such as nail atrophy, alopecia, gray hair, immunodeficiency and possibly also cancerogenesis. George Martin described the progerias as segmental diseases [66] and characterized their pathology and many molecular defects in pioneering investigations (summarized in reference [67]). In his original definition, each disease captures some, but not all, of the symptoms of aging, each with different (segmental) affected organ systems. The data described here support a more modular instead of a strictly segmental model, in which the aging phenotype is caused by a limited number of modules, as originally proposed by Hofer et al. [68], some of which are unique and some of which overlap the symptoms of other modules. TPE-OLD can provide an explanation for this model on a molecular basis. Depending on the degree of the telomere damage and disease-specific peculiarities, a phenotype variety may result with a more progeroid or more cancerogenic gene expression pattern.

## MATERIALS AND METHODS

### Cell cultures

The fibroblast cell lines were propagated in Amniomax medium with gentamicin sulfate and L-glutamine supplement. Lymphoblastoid cells (LCLs) were prepared and cultured as described [69].

### DNA samples

DNA was isolated from the blood of 27 NBS heterozygotes, 38 NBS homozygotes, a homozygous NBS fetus, and 108 controls. The NBS fetus presented with microcephaly, craniofacial dysmorphology, and signs of immaturity, but no malformation of inner organs. The pregnancy was interrupted in the 32^nd^ week.

### Molecular genetics

The founder mutation was analyzed as previously described [6]. Expression of the human telomerase reverse transcriptase gene (*hTERT*) was analyzed by qPCR. GAPDH was used as the internal control gene. The primer sequences for *hTERT* were: 5’- CCG ATT GTG AAC ATG GAC TAC GT -3’(forward); 5’- CGT AGT TGA GCA CGC TGA ACA -3’(reverse). The primer sequences for GAPDH were: 5’-CTC TGC TCC TCC TGT TCG AC-3’(forward); 5’-GCG CCC AAT ACG ACC AAA TC-3’(reverse).

mRNA quantification of TPE-OLD (telomere position effect over long distances) candidate genes was carried in triplicates in a 384-well plate using a BioRad CFX384 real-time C1000 thermal cycler, as described [70]. Human PPIA (Cyclophilin A) (Cat. 4333763F) was used as an endogenous control. The ΔΔCt method was used for relative quantification [70]. The following gene expression TaqMan assays (Applied Biosystems) were used: BSG (Cat. Hs00936295_m1), CACNA1A (Cat. Hs01579431_m1), COL5A3 (Cat. Hs01555669_m1), DDX39A (Cat. Hs01124952_g1), GAMT (Cat. Hs00355745_g1), NOTCH3 (Cat. Hs01128537_m1), OLFM2 (Cat. Hs01017934_m1), RNASEH2A (Cat. Hs00197370_m1), SCAMP4 (Cat. Hs00365263_m1), and UHRF1 (Cat. Hs01086727_m1). qPCR was applied to determine the relative telomere length as described previously [71]. The relative length of individual telomeres was analyzed by quantitative fluorescence in situ hybridization (Q-FISH) [72]. The fluorescence intensity of single telomeres *(T)* relative to a constant repetitive region in the centromeric region (C) of chromosome 2 (T/C ratio) was measured, using Ikaros software. The T/C ratio of 15 metaphases was estimated, and the mean telomere intensities of the p-arms and q-arms were calculated for each chromosome.

Absolute TL was measured by Terminal Restriction Fragment (TRF) length analysis, using the Roche -TeloTAGGG TL assay and the restriction enzymes Hinf1 and Rsa1 [28].

Humanized NBS mice, kindly provided by André Nussenzweig, were generated by reconstitution of *Nbn* knockout mice with the human *NBN* gene carrying the c.657_661del5 mutation or the wild type allele [28].

### hTERT immortalization

Fibroblasts were infected with retroviral supernatants from a packaging cell line (PA317-TERT) that stably expresses the human telomerase cloned into a pBabePuro vector, as described previously [73].

### Identification of TPE-OLD candidate genes in human fibroblasts

Using an Affymetrix cDNA microarray, we compared the expression profiles of pre-senescent fibroblasts and *hTERT* immortalized fibroblasts. As a model for stress-inducible senescence, we used UV-radiated cells (radiated twice per day, for 3 consecutive days, with a total dose of 528 J/m², using four 20W TL/12 lamps emitting broadband UV-B peaking at 312 nm). Senescence was confirmed by β-galactosidase staining. We analyzed 16 microarrays, each with 54,675 transcripts on an HGU133-A2.0 array from Affymetrix®, in a design with 3 groups. Differentially expressed genes in replicative senescence, up to 15 Mbp telomeric on p19, that were not differentially expressed in UV-B treated cells were viewed as potential TPE-OLD candidates.

### Analysis of caspase-7 in western blots

Lmyphoblastoid cells were treated in triplicate with 10 μg/ml bleomycin for 0h, 12h, 24h, and 48h. Caspase fragments were separated by SDS gel electrophoresis, using the primary antibody *cleaved Caspase-7* (Asp 198) and the second antibody (Anti-Mouse IgG Horsradish Peroxidase).

### Detection of ATM and phosphorylated ATM (p-ATM) by immunoprecipitation

For examination of phosphorylation of ATM after bleomycin treatment, lymyphoblastoid cells in logarithmic growth were treated in triplicate with 0, 10, and 30 μg/ml bleomycin for 1 h at 37°C. Immunoprecipitation was performed with the FISH-antibody (Anti-ATM, rabbit polyclonal, Novus) and magnetic beads (Dynal Biotech ASA/Invitrogen). The separated proteins were probed with anti-ATM pS1981 (Rockland, monoclonal, mouse), and reprobed with an anti-ATM antibody (Abcam Cambrige, UK) [74].

### Statistical analysis

The original data were exported to Excel 2007, GraphPad Prism 5 software, and SPSS 15.0 software for graphs and boxplots. The statistical tests used were Mann-Whitney U test, Fisher’s exact test, and the unpaired t-test.

### Analysis of chromosome fragility

Chromosome preparations were performed using standard techniques. The lymphoblastoid cell lines were analysed for chromosomal aberrations 4 h after irradiation (Muller MG 150 *x* ray apparatus; U_A_, 100 kV; I, 10 mA; filter, 0.3 mm Ni; dose rate, 2.1 Gy/min; Seifert, Hamburg, Germany) with 0 Gy, 0.5 Gy and 1.0 Gy including 1h colcemid treatment. 50 cells were analysed each. The types of aberrations were classified as achromatic lesions, chromatid and isochromatid breaks, and chromatid translocations. In order to calculate the total number of chromatid breaks per cell the latter were counted twice, the achromatic lesions and the telomere fusions neglected. Detailed protocols of the cytogenetic and molecular genetic methods are presented in [74].

### Ethics statement

All procedures were performed in accordance with the ethical standards of the responsible committee. The cell lines were established with the ethical approval of “The Children’s Memorial Health Institute, Warsaw”, the Ethics Committee of the Second Medical School of Charles University in Prague, and the consent of the subjects, or their parents (for children and the fetus). The mouse studies were approved by the State Office for Health and Social, Berlin (G0438/09).

### Data availability statement

The data that supports the findings of this study are available in the supplementary material of this article.

## Supplementary Material

Supplementary Figures

Supplementary Tables
