# Supplementary material for: Telomere attrition and dysfunction: a potential trigger of the progeroid phenotype in nijmegen breakage syndrome
Source: Aging (Albany NY). 2020 Jun 20;12(12):12342–75. doi: 10.18632/aging.103453 (PMC7343506; doi:10.18632/aging.103453)
Supplement: Supplementary Figures [file aging-12-103453-s002..pdf]

SUPPLEMENTARY FIGURES

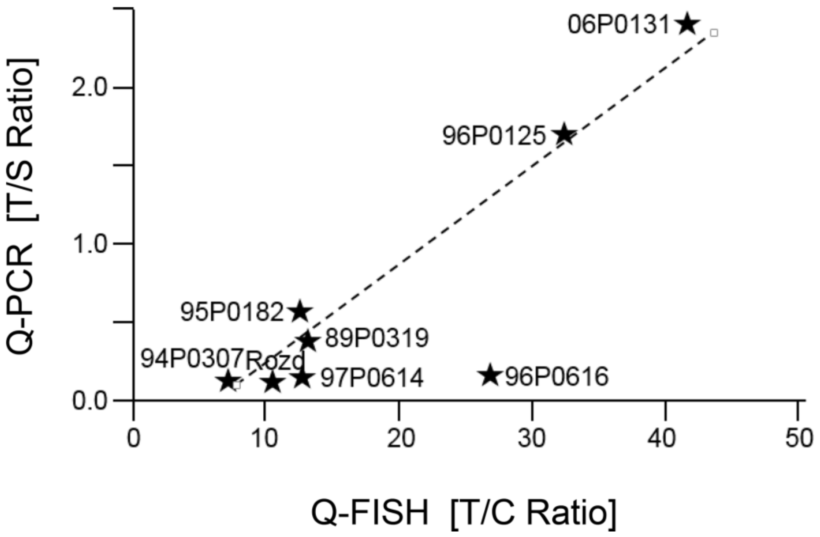

Supplementary Figure 1. Correlation between telomere length analyzed by qPCR and Q-FISH in six NBS lymphoblastoid cell lines and two controls (06P0131,96P0125). Original from [28].

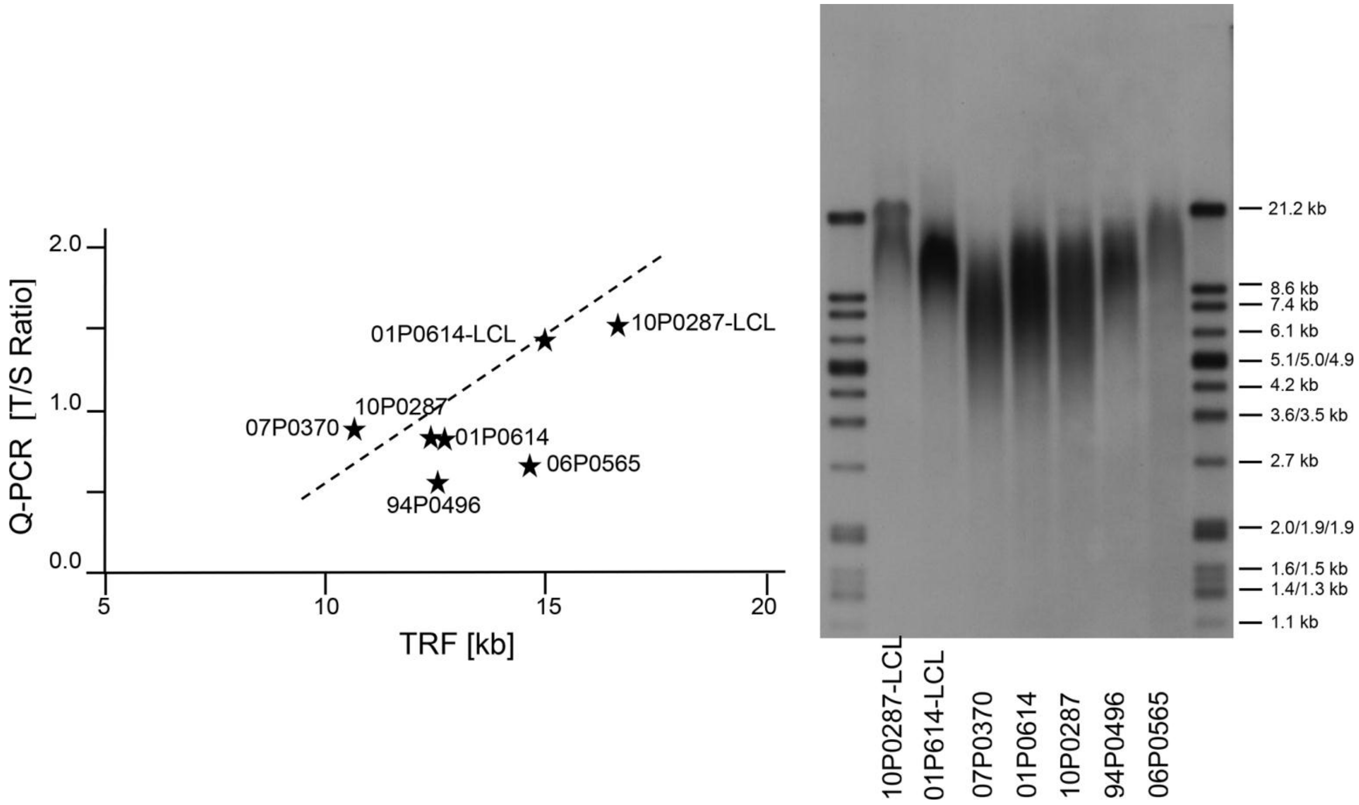

Supplementary Figure 2. Correlation of telomere length measured by qPCR and TRF analysis. The equation of the computed regression line is y= 3.4(X) +10.11 with a correlation coefficient of r= 0.64. Mean TRF length has been defined according to the formula described in Roche Application Note No. 12 209 136 001 (April 2018).

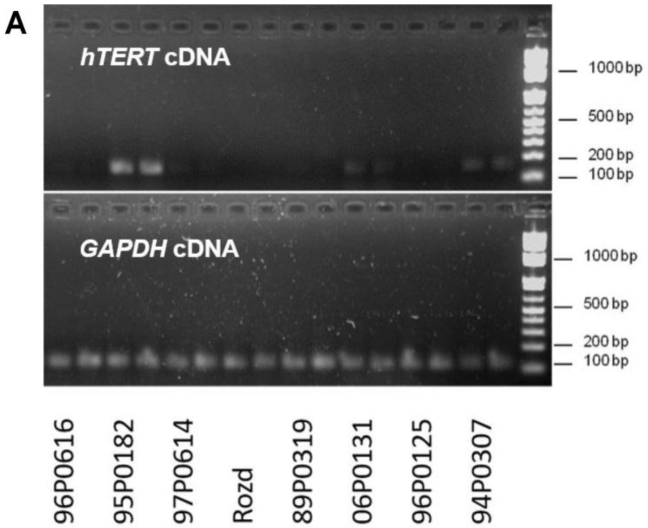
A
hTERT cDNA
GAPDH cDNA
1000 bp
500 bp
200 bp
100 bp
96P0616
95P0182
97P0614
Rozd
89P0319
06P0131
96P0125
94P0307

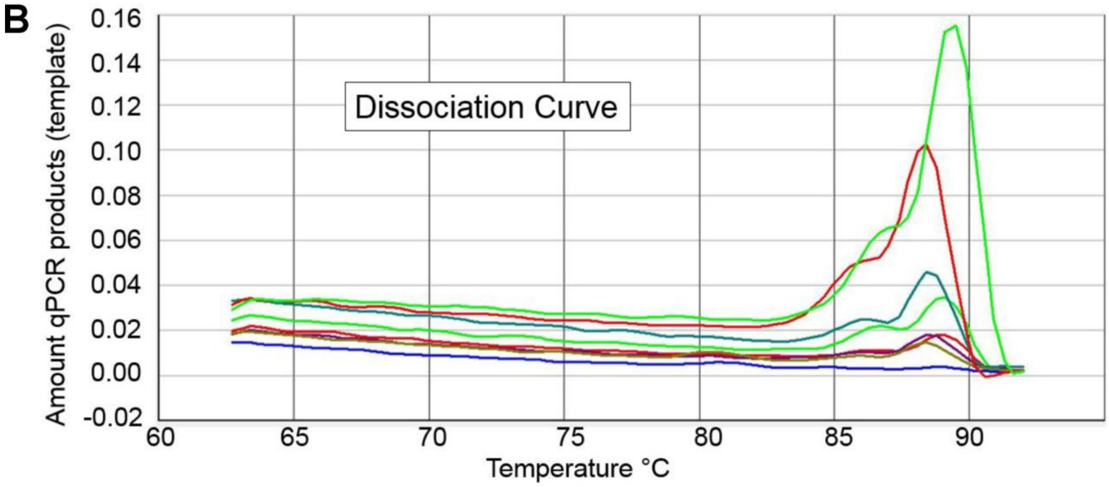
B
Dissociation Curve
Amount qPCR products (template)
0.16
0.14
0.12
0.10
0.08
0.06
0.04
0.02
0.00
-0.02
60
65
70
75
80
85
90
Temperature °C

C

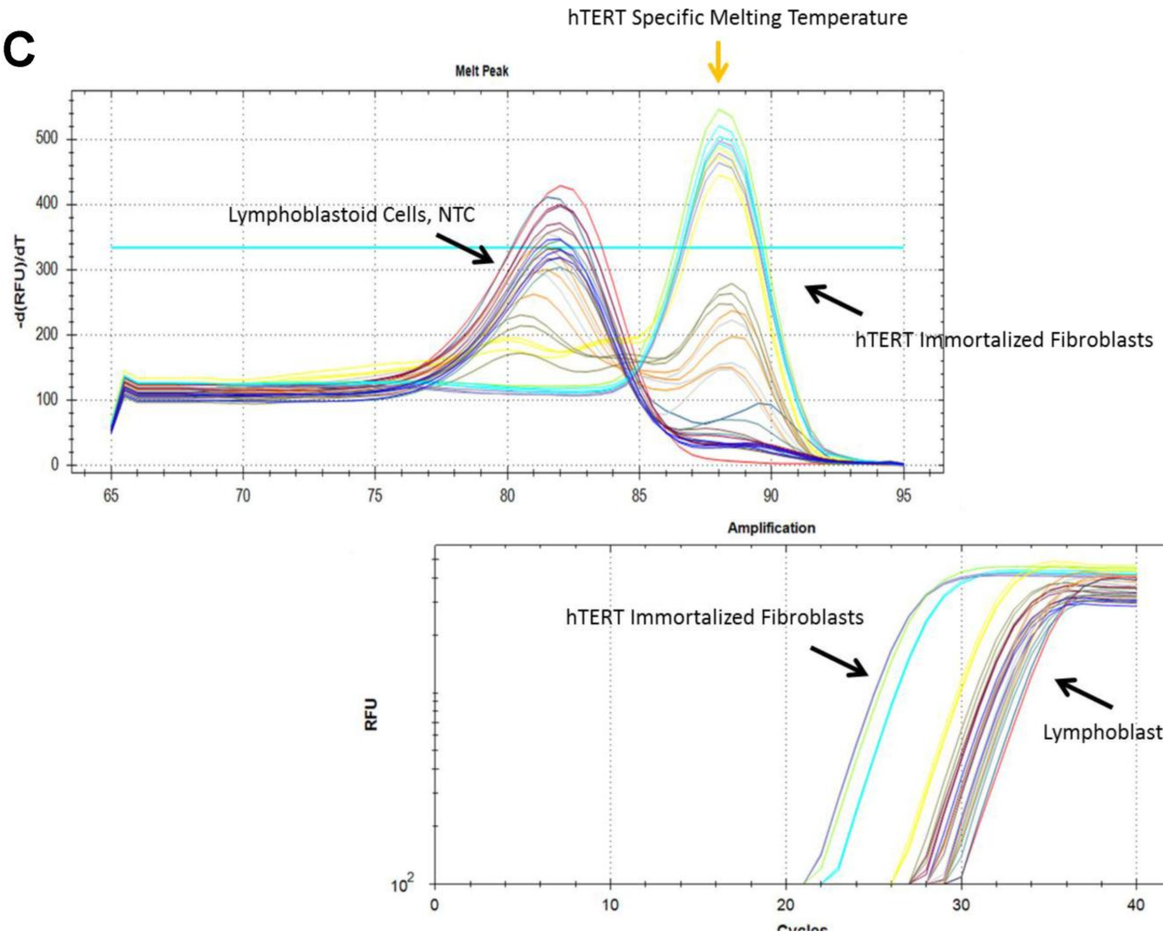

Supplementary Figure 3. (**A**) Expression of the *hTERT* gene in lymphoblastoid cell lines (LCLs) and *hTERT* immortalized fibroblasts. Agarose gel electroporesis of the PCR products of the *hTERT* cDNA and the GAPDH cDNA as internal control. 06P0131 and 96P0125 are LCL controls derived from male individuals homozygous for the wild type allele. Original from [28]. (**B**) Expression of *hTERT* as measured by qPCR. Dissociation curve of qPCR for *hTERT* cDNA (template). The high fluorescent peaks correspond to 95P0182 (green) and 94P0307 (red). The weak fluorescent peaks correspond to 06P0131 (dark green) and 97P0614 (light green). The lower lines (no template) correspond to 89P0319 (purple), 96P0616 (red), Rozd (brown) and 96P0125 (blue). (**C**) Expression of *hTERT* as measured by qPCR in a separate experiment. Dissociation curve of qPCR for *hTERT* cDNA (template) and non template control (NTC).

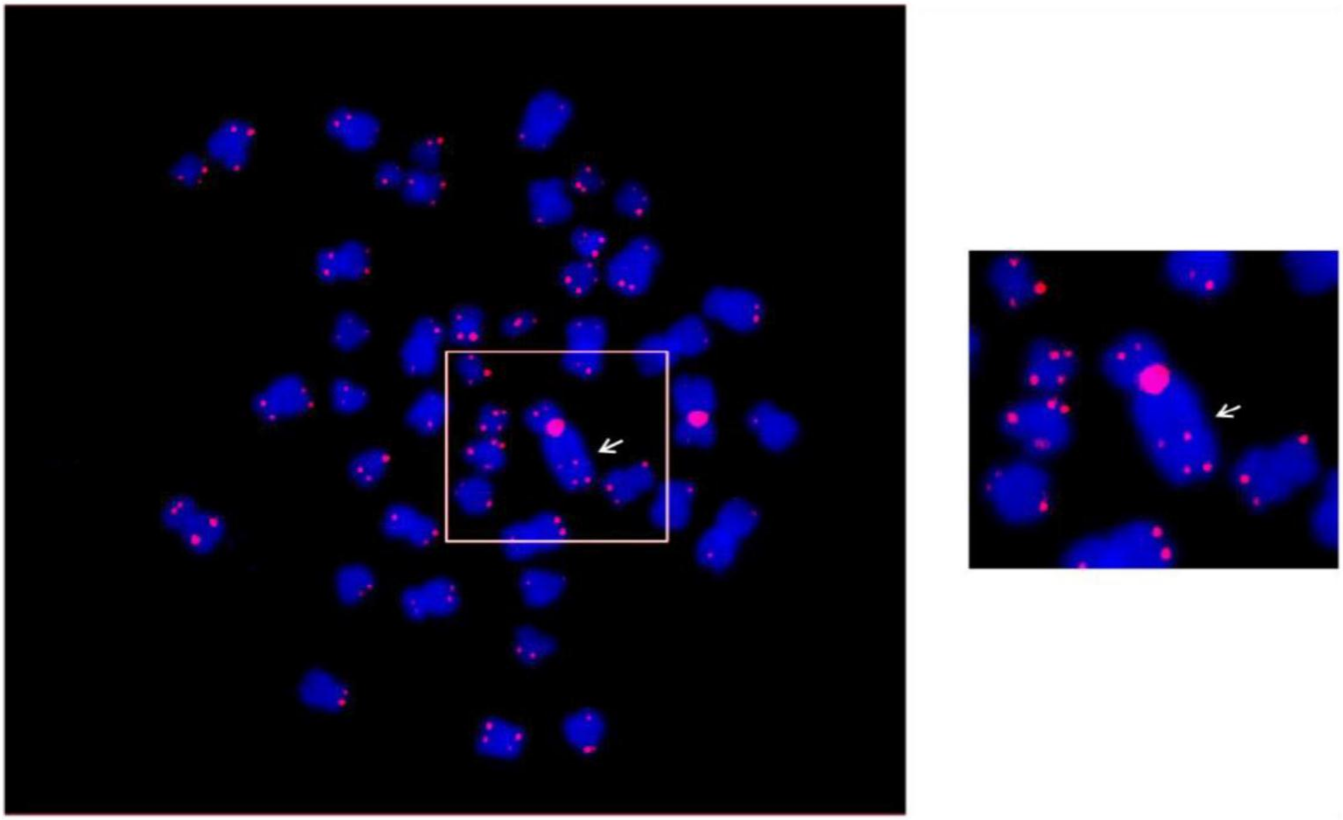

Supplementary Figure 4. Metaphase of NBS-LCL 94P0307 after Q-FISH. The arrow points to the telomere fusion between p telomere of chromosome 2 and an undefined chromosome.

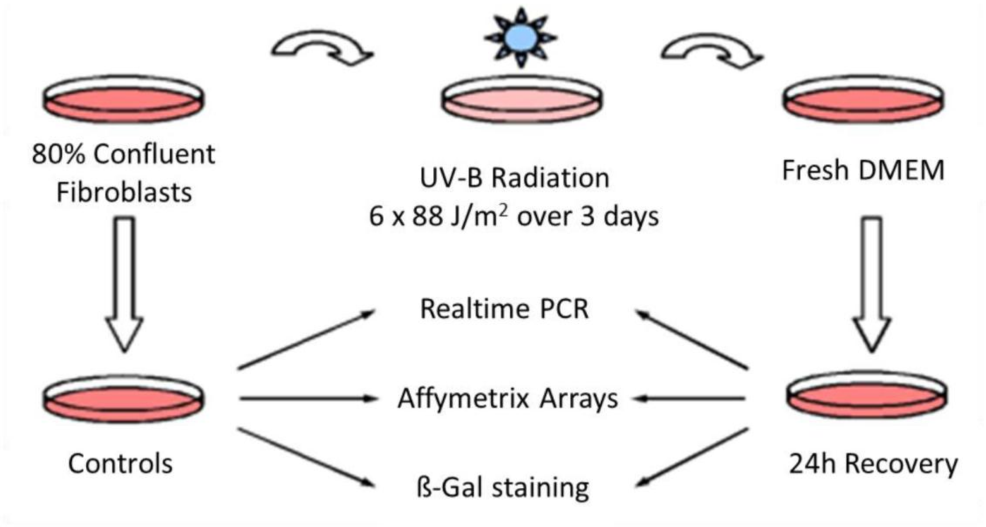

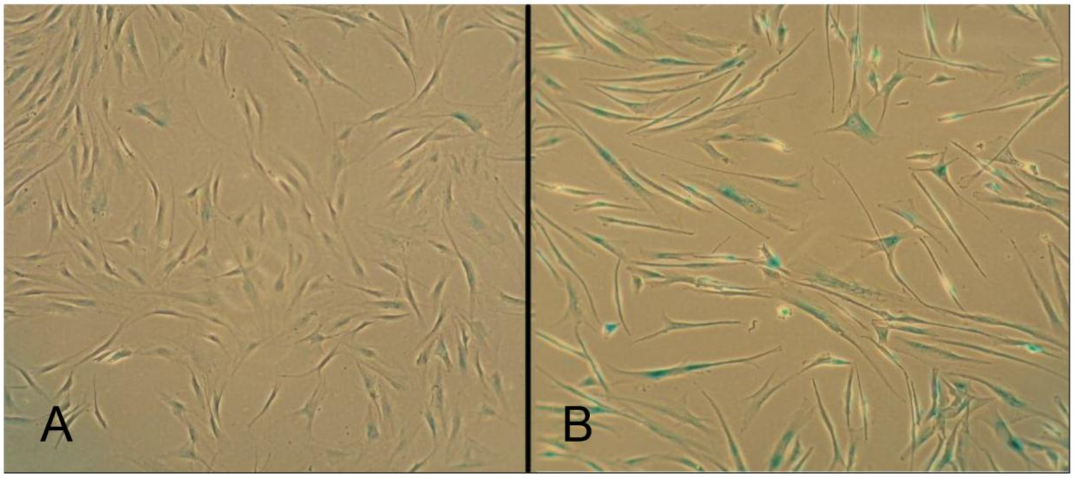

Supplementary Figure 5. TPE-OLD candidate gene approach - UV-Radiation. Human skin fibroblasts at 80 % confluence were exposed to UV-B radiation in a thin layer of DMEM using four 20W TL/12 lamps emitting broadband UV-B peaking at 312 nm. The procedure was performed twice a day for 3 consecutive days with a total dose of 528 J/m². 24 hours after the last irradiation analysis β galactosidase staining was performed. A, pre-treatment. B, senescent cells.

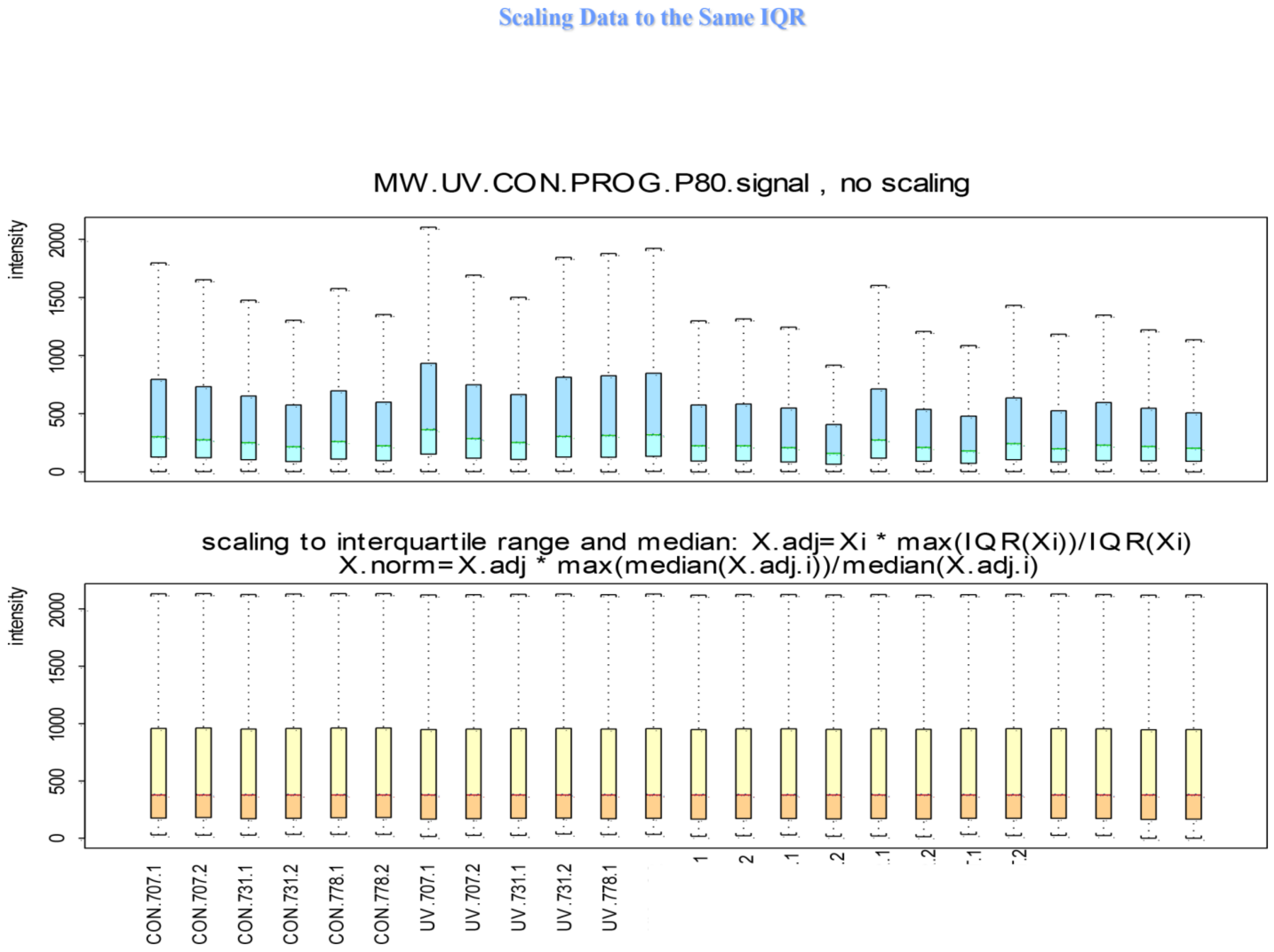

Supplementary Figure 6. TPE-OLD candidate gene approach – Statistical analysis. The unnormalized raw data of all experiments were exported in an ASCII format from the Affymetrix GCOS®-Software and imported in the statistical platform S-Plus 8.0® (Insightful Corporation, Seattle, Washington). Ten experiments were assigned two groups based on the characteristics of UV-treatment (6 repeats) and pre-senescence (4 repeats). Both groups were compared to six control experiments. The aim was to identify the most relevant genes discriminating between cells with UV-treatment and cells from pre-senescence, HGP). For this purpose a computer program based on S-Plus which computes the required subsets of genes and simultaneously controls the overall false positive rate was used. 16 microarrays were analyzed, each with 54.675 transcripts on an HGU133-A2.0 array from Affymetrix®, in a design with 3 groups, each with 4-6 replicates. To keep the data at a high quality level we used the GCOS Detection calls to discard all genes (transcripts) with callrates lower than 80% present in at least 13 of 16 repeats in a total of 19.984 transcripts. To make the data of different microarrays comparable, the Affymetrix-GCOS Signal-values were first normalized to have the same interquartile range (IQR) as the maximum IQR of the set and then each chip's median was shifted to the maximum median of the chip set.

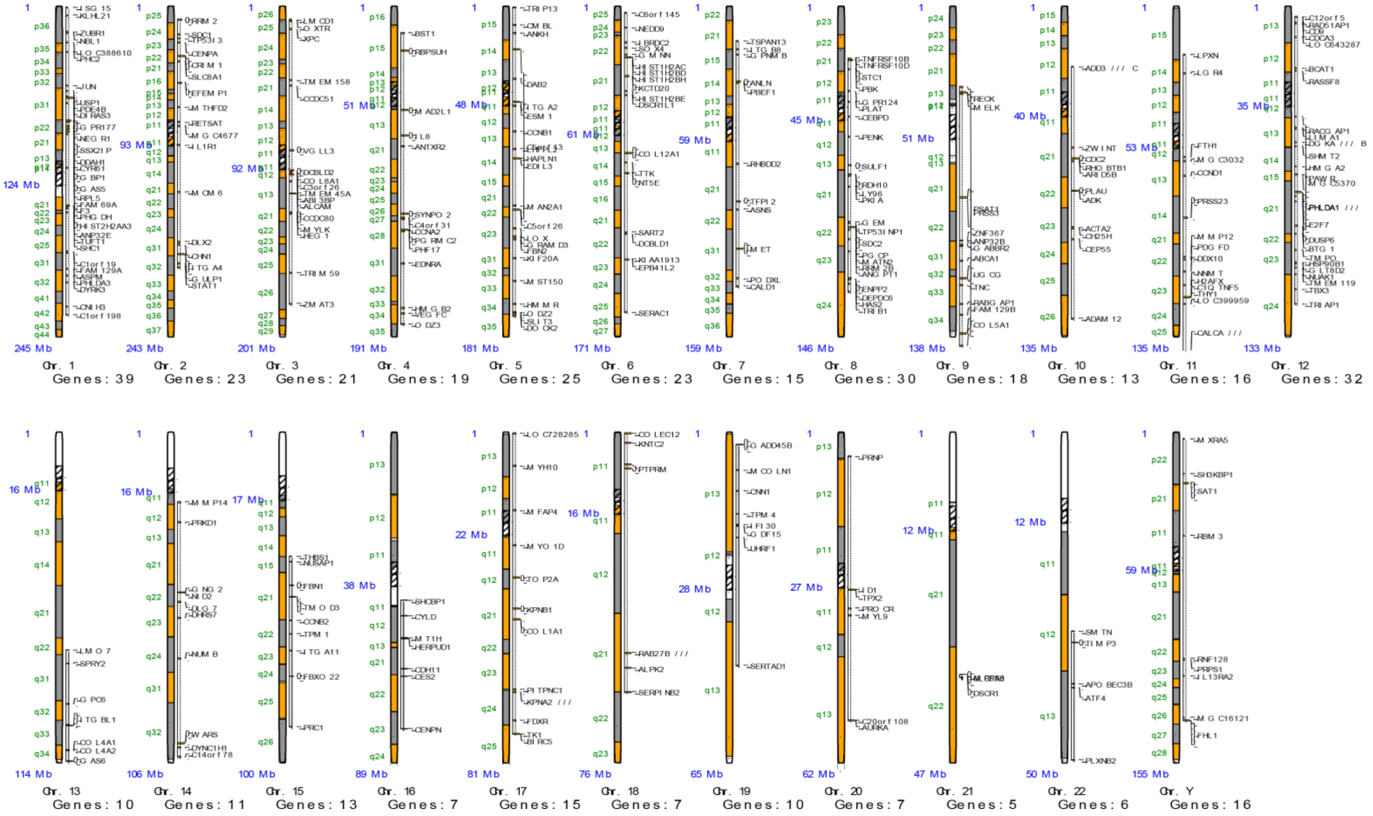

Supplementary Figure 7. TPE-OLD candidate gene approach. Differentially expressed genes (DEGs), dependent on chromosomal localization. For simplification only the DEGs with highest significance level are shown.

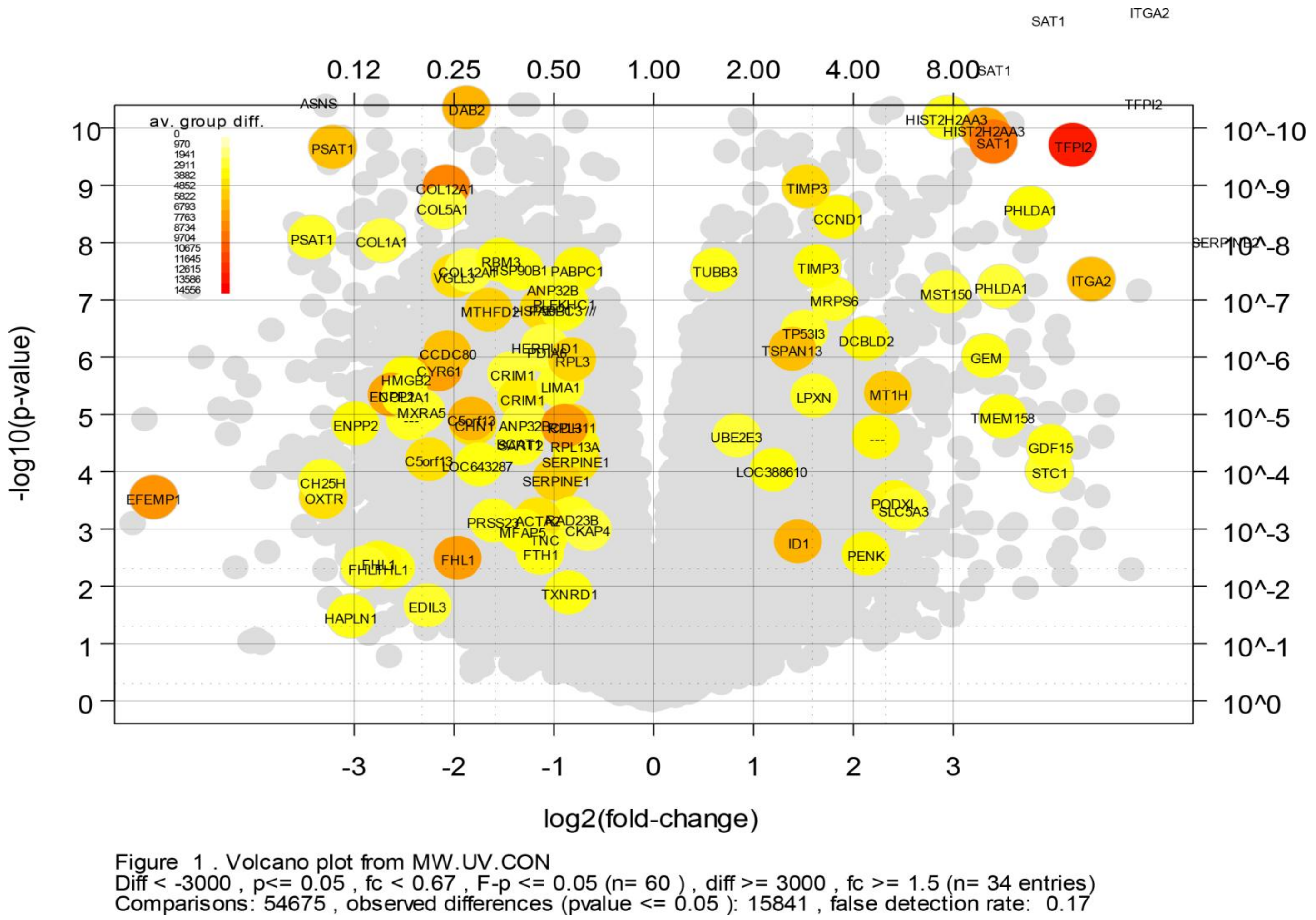

Supplementary Figure 8. TPE-OLD candidate gene approach. Differentially expressed genes (DEGs), visualized as Volcano Plot.
