# Supplementary material for: Telomere attrition and dysfunction: a potential trigger of the progeroid phenotype in nijmegen breakage syndrome
Source: Aging (Albany NY). 2020 Jun 20;12(12):12342–75. doi: 10.18632/aging.103453 (PMC7343506; doi:10.18632/aging.103453)
Supplement: Supplementary Tables [file aging-12-103453-s001..pdf]

SUPPLEMENTARY TABLES

Supplementary Table 1. Telomere length of healthy individuals (controls).

DNA number	relative telomere length	Age (years)	Sex
04P0523	2.16	1	F
04P0862	3.41	1	F
05P0586	1.6	2	M
05P0509	1.12	2	F
04P0535	1.45	3	F
01P0888	0.9	3	M
02P01053	2.25	5	M
04P0216	1.42	9	M
112	1.37	11	F
204	1.29	11	F
166	1.83	12	F
17	1.94	13	F
443	1.93	14	F
444	1.36	15	M
210	0.98	16	F
365	1.51	17	F
247	1.23	18	F
47	1.17	19	F
293	1.58	19	-
193	0.74	20	M
390	1.41	20	M
431	1.25	20	M
423	0.79	20	M
11	1.2	20	M
161	1	20	F
420	0.73	20	F
o2	1	21	M
440	1	21	F
143	1.26	21	F
448	0.83	21	F
129	0.93	21	F
169	1.16	21	F
164	1.18	21	F
13	1.22	21	M
413	0.9	21	F
223	1	22	F
163	0.95	22	M
40	0.91	22	F
188	0.76	22	F
241	1	23	M
246	0.84	23	M
470	1	23	M
280	1.44	23	F
262	0.99	23	F
191	0.89	23	F
328	0.78	24	M
333	1.14	24	F
471	1.39	24	F
464	0.75	24	M
284	1	24	F
150	1	24	F
330	0.85	24	F
292	1.26	24	M
o1	0.97	25	F

425	0.81	25	F
49	1.65	25	F
165	0.99	25	F
189	0.92	25	M
323	1.33	25	M
90	0.84	26	F
320	1.23	26	F
93	1.72	26	F
41	1.52	26	F
388	0.78	27	F
147	0.75	27	F
303	1.34	27	F
445	0.88	27	F
430	0.84	27	F
102	1.3	27	F
32	1	28	M
313	1.54	28	F
160	0.94	28	M
472	0.73	28	F
347	1	28	F
146	0.74	28	M
222	1.1	28	F
Ra	1	28	F
305	1.51	29	F
383	0.92	29	F
207	1	29	F
37	0.81	29	M
73	1.12	29	M
187	0.96	29	M
104	0.74	29	F
23	0.71	29	-
31	0.92	30	F
269	0.84	30	F
1	0.88	30	M
271	1.23	30	F
372	0.93	30	M
27	0.75	30	-
595	1.71	35	M
188	1.21	37	M
596	0.88	38	M
192	0.78	39	M
597	0.94	40	-
194	1	43	F
584	0.79	45	-
591	0.53	60	F
592	0.51	60	M
579	0.4	64	M
590	0.47	66	M
570	0.56	67	M
574	0.5	74	M
588	0.51	75	F
572	0.61	78	F
580	0.61	78	M
587	0.61	80	M

Age group	Number	Mean	SD	Max	Min
1-10	8	1.79	0.80	3.41	0.9
11-20	18	1.30	0.37	1.94	0.73
21-30	65	1.03	0.24	1.72	0.71
31-45	7	1.04	0.33	1.71	0.78
46-80	10	0.53	0.07	0.61	0.4

Supplementary Table 2. Estimation of telomere length of NBS-homozygotes.

DNA number	Relative telomere lengths	Age (years)	Sex
13720	1.62	1	M
12207	1.48	1	M
13424	1.29	1	F
6645	0.3	1	F
5342	0.21	1	M
6018	0.68	2	F
7105	0.44	2	F
3769	0.43	2	M
3772	0.43	2	F
7694	2.1	3	F
11766	2	3	F
8294	2.1	3	F
13607	0.98	5	F
13028	1.55	6	M
3426	1.35	6	F
8165	1	6	M
9990	1.3	6	M
11348	1.17	7	F
5202	0.67	7	F
3201	0.35	7	F
4530	0.22	7	F
8304	0.34	8	M
12557	1.13	8	-
5700	0.99	9	F
11154	1.73	10	M
7822	0.96	10	M
9028	0.78	10	F
5546	0.58	10	M
6023	1.3	12	F
13921	0.76	12	M
2552	0.69	13	M
3197	0.2	13	M
3205	1.3	14	M
3316	0.29	15	M
5567	0.64	15	M
5450	0.56	18	F
10376	0.46	19	F
5431	0.26	20	F

1-10 years:

Category	Number	Mean	SD	Max	Min
Control	8	1.79	0.80	3.41	0.90
NBS-Homozygote	28	1.01	0.58	2.10	0.21

11-20 years

Category	Number	Mean	SD	Max	Min
Control	18	1.30	0.37	1.94	0.73
NBS-Homozygote	10	0.65	0.39	1.30	0.20

Supplementary Table 3. Estimation of telomere length of NBS-heterozygotes.

DNA number	Relative telomere lengths	Age (years)	Sex
5343	2	1	F
10130	1.51	2	M
4328	0.89	3	M
8296	1.68	3	M
3221	1	4	M
3223	2	5	F
10133	1.38	5	F
10131	0.99	5	M
10123	2.35	7	M
7234	1.56	8	F
6017	1.77	8	M
3224	1.49	10	M
10120	0.71	12	F
4787	1.31	19	M
8295	0.71	22	F
3222	1	25	F
10124	2	25	M
508	1	27	F
6024	1.3	30	F
466	1.3	30	M
7235	0.79	30	M
7824	0.75	33	M
7823	0.78	35	M
6025	0.32	40	M
4786	0.25	40	M
464	0.6	50	F
463	0.27	54	M

Age	Category	Number	Mean	SD	Max	Min
1 -- 10	Control	8	1.79	0.80	3.41	0.90
	NBS-Heterozygote	12	1.55	0.45	2.35	0.89
11 -- 30	Control	83	1.08	0.29	1.94	0.71
	NBS-Heterozygote	9	1.12	0.41	2.00	0.71
>30	Control	17	0.74	0.33	1.71	0.40
	NBS-Heterozygote	6	0.50	0.24	0.78	0.25

Supplementary Table 4. Analysis of chromosome fragility after irradiation in six NBS lymphoblastoid cell lines with the absolute longest (above) and absolute shortest (below) telomere lengths.

	Survival (y)	Aberrant mitoses (%)			Breaks (%)		
		0 Gy	0,5 Gy	1,0 Gy	0 Gy	0,5 Gy	1,0 Gy
89P0319 ♀	0	14	69	88	0.16	1.42	2.2
96P0616 ♀	0,2	2	38	42	0.02	0.54	0.68
95P0182 ♀	2,8	0	42	100	0	1.18	3.5
**Mean ± SD**		**5 ± 8**	**50 ± 7**	**77 ± 31**	**0.16 ± 0,02**	**1.05 ± 0,45**	**2.13 ± 1,41**
97P0614 ♂	>12	2	40	42	0.04	0.52	0.66
RoZd ♀	>12	0	39	59	0	0.48	0.71
94P0307 ♂	>12	0	42	72	0	1.16	1.58
		**1 ± 1**	**40 ± 2**	**58 ± 15**	**0.01 ± 0.02**	**0.72 ± 0.38**	**0.98 ± 0.52**
**Control**		**0**	**16**	**10**	**0**	**0.2**	**0.14**

Chromatid breaks were distinguished from chromatid translocations. Since the latter are due to two breakage events, they were counted twice for the calculation of the total break events. Dicentric chromosomes were not scored because they cannot be induced in the G2 phase and apparently are due to telomere fusions.

Supplementary Table 5. T/C-FISH data on telomere lengths of NBS-LCL 94P0307.

Chromosome	Median (± SD) of p-arm	Median (± SD) of q-arm
1	8.7 (±4.8)	8 (±5.9)
2	5.2 (±4.5)	5.1(±8.3)
3	9.0 (±9.3)	9.9 (±6.1)
4	11.2(±10.1)	7.2(±10.5)
5	9.1(±5.7)	11.4(±11.1)
6	9.1(±7.1)	7.8(±4.8)
7	10.6(±6.1)	4.9(±6.7)
8	9.3 (±6.3)	7.1(±6.6)
9	10.7(±5.9)	7.3(±5.5)
10	10.2(±9.4)	17.4(±11.1)
11	9.5(±6.5)	7 (±5.6)
12	8.9(±5.2)	7.5(±5.4)
13	5.6(±6.1)	8.6(±7.3)
14	6.4(±7.3)	9.8(±6.9)
15	5.5(±4.5)	9.7(±4.9)
16	7.5(±6.5)	8.1(±5.1)
17	5 (±4.6)	6.1(±5.7)
18	6.9(±6.2)	5.8(±6.6)
19	13.2(±36.6)	6.2(±5.9)
20	5.3(±4.2)	6.7(±4.1)
21	4.6(±3.2)	6.2(±6.1)
22	5.7(±3.5)	4.9(±3.3)
X	6.6(±7.1)	6.1(±7.9)
Y	6.3(±3.3)	4.5(±5.1)

Supplementary Table 6. Q-FISH analysis of telomere lengths of the p arm of chromosome 19 in 11 metaphases of the NBS lymphoblastoid cell line 94P0307. T/C values are presented for the highly and weakly fluorescent chromosome 19.

Metaphase	1	2	3	4	5	6	7	8	9	10	11	⌀
T/C value: high	85.2	52.6	47.1	44.1	60.5	33.4	79.4	60.2	32.9	68.3	58.6	56.6
T/C value: low	3.2	12.6	2.0	2.1	8.2	1.1	2.1	6.3	2.2	3.5	9.6	4.8

Supplementary Table 7A. TPE-OLD candidate gene approach using Affymetrix expression data in senescent (HGP), *hTERT* immortalized senescent and UV-B-treated fibroblasts for comparison. In yellow those genes with highly significant expression changes in senescent but not in UV-B-treated and/or not in *hTERT* immortalized senescent fibroblasts.

iReports Results

Gene Symbol (iRepor	Chromosome locatio	Gene description	c=changed, ---=known, u=unknown)	Gene Symbol (Affymetrix)	Probe Set ID	HGP vs. CON: Gene Symbol (Affymetrix)	P-Value	Entrez Gene I	Fold Chang	Probe Set ID	Source
BSG	571,297-583,493		---	BSG	208677_s_at	BSG	0,0000001809	682	2,840	208677_s_a	HGP
BTBD2	1,985,447-2,034,880		---	BTBD2	207722_s_at	BTBD2	0,0000003871	55643	2,024	207722_s_a	HGP
CACNA1A	13,317,256-13,734,804		---	CACNA1A	206399_x_at	CACNA1A	0,0001904053	773	2,096	206399_x_a	HGP
COL5A3	10,070,237-10,121,147		---	COL5A3	218975_at	COL5A3	0,0207965844	50509	2,367	218975_at	HGP
DDX39A	14,519,610-14,530,195	DEAD (Asp-	c	DDX39	201584_s_at	DDX39	0,0000313020	10212	-2,033	201584_s_a	HGP
FKBP8	18,531,751-18,544,077		---	FKBP8	208255_s_at	FKBP8	0,0000000564	23770	3,060	208255_s_a	HGP
FKBP8	18,531,751-18,544,077		---	FKBP8	40850_at	FKBP8	0,0000002468	23770	2,710	40850_at	HGP
GAMT	1,397,088-1,401,569		---	GAMT	205354_at	GAMT	0,0000137147	2593	2,071	205354_at	HGP
GAMT	1,397,088-1,401,569		---	GAMT	1552474_a_at	GAMT	0,0000009301	2593	2,006	1552474_a_	HGP
ICAM1	10,381,511-10,397,291		---	ICAM1	202638_s_at	ICAM1	0,0068949400	3383	3,355	202638_s_a	HGP
ICAM1	10,381,511-10,397,291		---	ICAM1	202637_s_at	ICAM1	0,0015622485	3383	2,182	202637_s_a	HGP
ISYNA1	18,545,198-18,549,111		---	ISYNA1	222240_s_at	ISYNA1	0,0004248998	51477	2,104	222240_s_a	HGP
JUND	18,390,563-18,392,432		---	JUND	203751_x_at	JUND	0,0000014634	3727	2,581	203751_x_a	HGP
JUND	18,390,563-18,392,432		---	JUND	214326_x_at	JUND	0,0000020790	3727	2,695	214326_x_a	HGP
MAP2K2	4,090,319-4,124,126		---	MAP2K2	213490_s_at	MAP2K2	0,0000007541	5605	2,068	213490_s_a	HGP
MARCH2	8,478,154-8,503,901		---	MARCH2	210075_at	MARCH2	0,0001449725	51257	2,193	210075_at	HGP
NOTCH3	15,159,038-15,200,981		---	NOTCH3	203238_s_at	NOTCH3	0,0112953135	4854	4,109	203238_s_a	HGP
OLFM2	137,967,268-138,013,025		---	OLFM2	223601_at	OLFM2	0,0066981369	93145	3,824	223601_at	HGP
PALM	708,953-748,330		---	PALM	203859_s_at	PALM	0,0023807474	5064	3,551	203859_s_a	HGP
RAB11B	8,454,865-8,469,318		---	RAB11B	34478_at	RAB11B	0,0000104773	9230	2,245	34478_at	HGP
RNASEH2A	12,917,394-12,924,462		---	RNASEH2A	203022_at	RNASEH2A	0,0005021651	10535	-2,063	203022_at	HGP
SCAMP4	1,905,213-1,926,016		---	SCAMP4	213244_at	SCAMP4	0,0000004595	113178	2,707	213244_at	HGP
SCAMP4	1,905,213-1,926,016		---	SCAMP4	235073_at	SCAMP4	0,0000035546	113178	2,345	235073_at	HGP
SLC44A2	10,713,121-10,755,235		---	SLC44A2	225175_s_at	SLC44A2	0,0005098295	57153	2,045	225175_s_a	HGP
SYDE1	15,218,214-15,225,799		---	SYDE1	216272_x_at	SYDE1	0,0027395466	85360	2,014	216272_x_a	HGP
SYDE1	15,218,214-15,225,799		---	SYDE1	212962_at	SYDE1	0,0012149194	85360	2,699	212962_at	HGP
SYDE1	15,218,214-15,225,799		---	SYDE1	216271_x_at	SYDE1	0,0005825874	85360	2,208	216271_x_a	HGP
UHRF1	4,903,092-4,962,165		---	UHRF1	225655_at	UHRF1	0,0000692885	29128	-2,653	225655_at	HGP
XAB2	7,684,411-7,694,451		---	XAB2	218110_at	XAB2	0,0000618183	56949	2,160	218110_at	HGP

Gene Symbol (iRepor	HGP-TERT vs. CON: Gene Symbol (Affymetrix)	P-Value	Entrez Gene I	Fold Chang	Probe Set ID	Source	UV vs. CON: Gene Symbol (Affymetrix)	P-Value	Entrez Gene I	Fold Chang	Probe Set ID	Source
BSG	BSG	0,0000076921	682	2,123	208677_s_a	HGP-TERT						
BTBD2	BTBD2	0,0000042508	55643	2,436	207722_s_a	HGP-TERT						
CACNA1A												
COL5A3												
DDX39A												
FKBP8	FKBP8	0,0000458219	23770	2,616	208255_s_a	HGP-TERT						
FKBP8	FKBP8	0,0000205907	23770	3,075	40850_at	HGP-TERT						
GAMT												
GAMT												
ICAM1	ICAM1	0,0045024336	3383	2,718	202638_s_a	HGP-TERT						
ICAM1												
ISYNA1							ISYNA1	0,0000632685	51477	3,151	222240_s_a	UV
JUND	JUND	0,0000122462	3727	2,480	203751_x_a	HGP-TERT						
JUND	JUND	0,0000132107	3727	2,780	214326_x_a	HGP-TERT						
MAP2K2	MAP2K2	0,0000168467	5605	2,866	213490_s_a	HGP-TERT						
MARCH2	MARCH2	0,0003620400	51257	2,099	210075_at	HGP-TERT						
NOTCH3												
OLFM2												
PALM	PALM	0,0034745851	5064	2,405	203859_s_a	HGP-TERT						
RAB11B	RAB11B	0,0020523284	9230	2,312	34478_at	HGP-TERT						
RNASEH2A												
SCAMP4	SCAMP4	0,0000100484	113178	2,738	213244_at	HGP-TERT						
SCAMP4												
SLC44A2	SLC44A2	0,0009311980	57153	2,094	225175_s_a	HGP-TERT						
SYDE1												
SYDE1	SYDE1	0,0104646032	85360	2,060	212962_at	HGP-TERT						
SYDE1												
UHRF1							UHRF1	0,0000024303	29128	-4,123	225655_at	UV
XAB2	XAB2	0,0000488575	56949	2,306	218110_at	HGP-TERT						

Supplementary Table 7B. TPE-OLD candidate gene approach using Affymetrix expression data in senescent cells, *hTERT* immortalized senescent cells and UV-B-treated cells, Raw data.

Raw data of Affymetrix experiments

SEN vs. CON

	in HGP, HGP-TERT & UV?	RowNames	Fold Chang	SIG	Gene.Symbol	Physical.Position	UniGene.ID
9	0	208677_s_a	2,484	****	BSG	572564	Hs.501293
10	0	207722_s_a	2,213	****	BTBD2	1985446	Hs.465543
11	0	206399_x_a	1,432	**	CACNA1A	13318114	Hs.501632
14	0	218975_at	3,604	*	COL5A3	10070236	Hs.235368
15	0	201584_s_a	-1,806	***	DDX39	14519626	Hs.311609
16	0	208255_s_a	4,278	****	FKBP8	18642572	Hs.173464
17	0	40850_at	3,360	****	FKBP8	18642572	Hs.173464
18	0	205354_at	2,434	****	GAMT	1397087	Hs.81131
19	0	1552474_a_	2,191	****	GAMT	1398451	Hs.81131
20	0	202638_s_a	3,470	**	ICAM1	10381778	Hs.643447
21	0	202637_s_a	3,260	*	ICAM1	10381763	Hs.643447
22	0	222240_s_a	3,428	***	ISYNA1	18545631	Hs.724523
23	0	203751_x_a	3,096	****	JUND	18390570	Hs.2780
24	0	214326_x_a	2,945	****	JUND	18391013	Hs.2780
26	0	213490_s_a	1,820	****	MAP2K2	128766471	Hs.465627
27	0	210075_at	2,496	***	MARCH2	8486669	Hs.631861
30	0	203238_s_a	4,695	**	NOTCH3	15270444	Hs.8546
31	0	223601_at	9,557	*	OLFM2	9964399	Hs.169743
32	0	203859_s_a	2,897	**	PALM	709001	Hs.631841
37	0	34478_at	2,002	***	RAB11B	8454778	Hs.626404
38	0	203022_at	-2,272	**	RNASEH2A	12917438	Hs.532851
39	0	213244_at	2,225	****	SCAMP4	1925044	Hs.144980
40	0	235073_at	1,723	**	SCAMP4	1905405	Hs.144980
41	0	225175_s_a	1,863	**	SLC44A2	10754489	Hs.534560
42	0	216272_x_a	2,756	*	SYDE1	15223120	Hs.706845
43	0	212962_at	2,645	**	SYDE1	15220561	Hs.528701
44	0	216271_x_a	2,217	**	SYDE1	15218244	Hs.528701
47	0	225655_at	-2,371	**	UHRF1	20704503	Hs.108106
48	0	218110_at	2,304	****	XAB2	7684411	Hs.9822

SEN-TERT vs. CON

	RowNames	Fold Chang	SIG	Gene.Symbol	pq	Chromosome	Physical.Position	UniGene.ID
9	208677_s_a	-1,203	**	BSG	19p13.3	19	572564	Hs.501293
10	207722_s_a	-1,030	n	BTBD2	19p13.3	19	1985446	Hs.465543
11	206399_x_a	1,082	n	CACNA1A	19p13.2-p1:	19	13318114	Hs.501632
14	218975_at	-1,417	n	COL5A3	19p13.2	19	10070236	Hs.235368
15	201584_s_a	1,356	n	DDX39	19p13.12	19	14519626	Hs.311609
16	208255_s_a	1,004	n	FKBP8	19p12	19	18642572	Hs.173464
17	40850_at	1,088	n	FKBP8	19p12	19	18642572	Hs.173464
18	205354_at	1,423	*	GAMT	19p13.3	19	1397087	Hs.81131
19	1552474_a_	1,286	n	GAMT	19p13.3	19	1398451	Hs.81131
20	202638_s_a	-1,632	n	ICAM1	19p13.3-p1:	19	10381778	Hs.643447
21	202637_s_a	-1,635	n	ICAM1	19p13.3-p1:	19	10381763	Hs.643447
22	222240_s_a	-1,022	n	ISYNA1	19p13.11	19	18545631	Hs.724523
23	203751_x_a	1,010	n	JUND	19p13.2	19	18390570	Hs.2780
24	214326_x_a	-1,007	n	JUND	19p13.2	19	18391013	Hs.2780
26	213490_s_a	1,191	n	MAP2K2	19p13.3	7	128766471	Hs.465627
27	210075_at	-1,041	n	MARCH2	19p13.2	19	8486669	Hs.631861
30	203238_s_a	-1,380	n	NOTCH3	19p13.2-p1:	19	15270444	Hs.8546
31	223601_at	-1,241	n	OLFM2	19p13.2	19	9964399	Hs.169743
32	203859_s_a	-1,394	n	PALM	19p13.3	19	709001	Hs.631841
37	34478_at	1,022	n	RAB11B	19p13.2	19	8454778	Hs.626404
38	203022_at	1,689	*	RNASEH2A	19p13.2	19	12917438	Hs.532851
39	213244_at	-1,058	n	SCAMP4	19p13.3	19	1925044	Hs.144980
40	235073_at	1,097	n	SCAMP4	19p13.3	19	1905405	Hs.144980
41	225175_s_a	1,535	**	SLC44A2	19p13.1	19	10754489	Hs.534560
42	216272_x_a	1,194	n	SYDE1	19p13.12	19	15223120	Hs.706845
43	212962_at	1,084	n	SYDE1	19p13.12	19	15220561	Hs.528701
44	216271_x_a	-1,077	n	SYDE1	19p13.12	19	15218244	Hs.528701
47	225655_at	2,404	**	UHRF1	19p13.3	12	20704503	Hs.108106
48	218110_at	-1,194	n	XAB2	19p13.2	19	7684411	Hs.9822

UV vs. CON

	RowNames	Fold Chang	SIG	Gene.Symbol	pq	Chromosome	Physical.Position	UniGene.ID
9	208677_s_a	1,377	***	BSG	19p13.3	19	572564	Hs.501293
10	207722_s_a	1,251	n	BTBD2	19p13.3	19	1985446	Hs.465543
11	206399_x_a	1,054	n	CACNA1A	19p13.2-p1:	19	13318114	Hs.501632
14	218975_at	-1,450	n	COL5A3	19p13.2	19	10070236	Hs.235368
15	201584_s_a	1,099	n	DDX39	19p13.12	19	14519626	Hs.311609
16	208255_s_a	1,065	n	FKBP8	19p12	19	18642572	Hs.173464
17	40850_at	1,131	n	FKBP8	19p12	19	18642572	Hs.173464
18	205354_at	2,035	****	GAMT	19p13.3	19	1397087	Hs.81131
19	1552474_a_	1,902	****	GAMT	19p13.3	19	1398451	Hs.81131
20	202638_s_a	1,553	*	ICAM1	19p13.3-p1:	19	10381778	Hs.643447
21	202637_s_a	-1,035	n	ICAM1	19p13.3-p1:	19	10381763	Hs.643447
22	222240_s_a	5,784	**	ISYNA1	19p13.11	19	18545631	Hs.724523
23	203751_x_a	1,690	****	JUND	19p13.2	19	18390570	Hs.2780
24	214326_x_a	1,907	****	JUND	19p13.2	19	18391013	Hs.2780
26	213490_s_a	1,163	n	MAP2K2	19p13.3	7	128766471	Hs.465627
27	210075_at	1,430	*	MARCH2	19p13.2	19	8486669	Hs.631861
30	203238_s_a	1,407	n	NOTCH3	19p13.2-p1:	19	15270444	Hs.8546
31	223601_at	1,195	n	OLFM2	19p13.2	19	9964399	Hs.169743
32	203859_s_a	1,201	n	PALM	19p13.3	19	709001	Hs.631841
37	34478_at	1,176	n	RAB11B	19p13.2	19	8454778	Hs.626404
38	203022_at	-1,906	**	RNASEH2A	19p13.2	19	12917438	Hs.532851
39	213244_at	1,313	*	SCAMP4	19p13.3	19	1925044	Hs.144980
40	235073_at	1,296	n	SCAMP4	19p13.3	19	1905405	Hs.144980
41	225175_s_a	1,040	n	SLC44A2	19p13.1	19	10754489	Hs.534560
42	216272_x_a	1,010	n	SYDE1	19p13.12	19	15223120	Hs.706845
43	212962_at	-1,137	n	SYDE1	19p13.12	19	15220561	Hs.528701
44	216271_x_a	-1,320	n	SYDE1	19p13.12	19	15218244	Hs.528701
47	225655_at	-4,345	****	UHRF1	19p13.3	12	20704503	Hs.108106
48	218110_at	-1,439	*	XAB2	19p13.2	19	7684411	Hs.9822

	Gene.Title	Probe Set ID	UniGene ID	Alignment	C
9	basigin (Ok	208677_s_a	Hs.501293	chr19:57256	b
10	BTB (POZ) d	207722_s_a	Hs.465543	chr19:19854	B
11	calcium ch	206399_x_a	Hs.501632	chr19:13318	c
14	collagen, ty	218975_at	Hs.235368	chr19:10070	c
15	DEAD (Asp-	201584_s_a	Hs.311609	chr19:14519	D
16	FK506 bindi	208255_s_a	Hs.173464	chr19:18642	F
17	FK506 bindi	40850_at	Hs.173464	chr19:18642	F
18	guanidinoa	205354_at	Hs.81131	chr19:13970	g
19	guanidinoa	1552474_a_	Hs.81131	chr19:13984	g
20	intercellular	202638_s_a	Hs.643447	chr19:10381	ir
21	intercellular	202637_s_a	Hs.643447	chr19:10381	ir
22	inositol-3-ph	222240_s_a	Hs.724523	chr19:18545	ir
23	jun D proto	203751_x_a	Hs.2780	chr19:18390	ju
24	jun D proto	214326_x_a	Hs.2780	chr19:18391	ju
26	mitogen-ac	213490_s_a	Hs.465627	chr7:128766	m
27	membrane	210075_at	Hs.631861	chr19:84866	m
30	Notch hom	203238_s_a	Hs.8546	chr19:15270	n
31	olfactomed	223601_at	Hs.169743	chr19:99643	o
32	paralemmi	203859_s_a	Hs.631841	chr19:70900	p
37	RAB11B, me	34478_at	Hs.626404	chr19:84547	R
38	ribonucleas	203022_at	Hs.532851	chr19:12917	ri
39	secretory c	213244_at	Hs.144980	chr19:19250	se
40	secretory c	235073_at	Hs.144980	chr19:19054	se
41	solute carri	225175_s_a	Hs.534560	chr19:10754	sc
42	synapse de	216272_x_a	Hs.706845	chr19:15223	sy
43	synapse de	212962_at	Hs.528701	chr19:15220	sy
44	synapse de	216271_x_a	Hs.528701	chr19:15218	sy
47	ubiquitin-lik	225655_at	Hs.108106	chr12:20704	u
48	XPA binding	218110_at	Hs.9822	chr19:76844	X

Supplementary Table 8. Regulation of telomeric 19p genes in normal and NBS fibroblasts with short telomeres (relative to cells with long telomeres).

Gene	Functions in senescence and/or cell growth	Reference	Distance to telomere [Mbp]	Differential gene expression, Affymetrix data	Differential gene expression, qPCR data Control fibroblasts	Differential gene expression, qPCR data NBS fibroblasts	TPE-OLD Effect
				[fold-change]			
BSG	MMP and cytokine production	[32]	0.6	+2.8	+3.2	+2.4	(N)
GAMT	ATP production (SASP?)	[33, 34]	1.4	+2.0	+5.6	+1.9	(N)
SCAMP4	player in SASP	[35]	1.9	+2.3	+1.9	+1.6	(N)
OLFM2	cell differentiation	[36]	9.9	+3.8	+50	+3.3	(N)
UHRF1	neg. regulator of senescence	[40]	4.9	-2.7	-5.0	+1.7	R
COL5A3	age-dependent tissue remodel.	[37]	10.0	+2.4	+25.0	+4.0	(N)
RNASEH2A	migration and invasion	[41]	12.8	-2.1	-4.5	+1.2	R
CACNA1A	calcium entry; nerve function	[38]	13.6	+2.1	+1.3	-1.5	R
DDX39A	role in cancer and longevity	[42]	14.6	-2.0	-2.2	+1.7	R
NOTCH3	pos. regulator of senescence (p21)	[39]	15.2	+4.1	+100.0	+58.0	(N)

BSG, basigin; GAMT, guanidinoacetate methyl-transferase; SCAMP4, secretory carrier membrane protein 4; OLFM2, olfactomedin 2; UHRF1, Ubiquitin like with PHD and ring finger domain 1; COL5A3, collagen type V alpha 3 chain; RNASEH2A, ribonuclease H2 subunit A; CACNA1A, calcium voltage-gated channel subunit alpha1 A; DDX39A, DExD-box helicase 39A; NOTCH3, Notch Receptor 3; SASP, senescence associated secretory phenotype; MMP, matrix metallopeptidases; (N), TPE-OLD: normal but attenuated regulation; R, TPE-OLD: reversed regulation, compared to regulation (mRNA) in healthy control fibroblasts; grey overlay: upregulation in pre-senescent cells; green overlay, down regulation in pre-senesecent cells.

Supplementary Table 9. Correlation between telomere length and clinical data of 21 NBS homozygotes.

Telomere length	Age at cancer manifestation	Age at death
	p	p
Long vs.median	0.07	0.50
Median vs. short	0.52	0.54
Long vs. short	0.41	0.99

The p values are based on the unpaired T- test.
